# Mono-, Di- and Tetra-iron Complexes with Selenium or Sulphur Functionalized Vinyliminium Ligands: Synthesis, Structural Characterization and Antiproliferative Activity

**DOI:** 10.3390/molecules25071656

**Published:** 2020-04-03

**Authors:** Gabriele Agonigi, Lucinda K. Batchelor, Eleonora Ferretti, Silvia Schoch, Marco Bortoluzzi, Simona Braccini, Federica Chiellini, Lorenzo Biancalana, Stefano Zacchini, Guido Pampaloni, Biprajit Sarkar, Paul J. Dyson, Fabio Marchetti

**Affiliations:** 1 Dipartimento di Chimica e Chimica Industriale, Università di Pisa, Via G. Moruzzi 13, I-56124 Pisa, Italy; g.ago2@hotmail.it (G.A.); silvia.schoch@phd.unipi.it (S.S.); simona.braccini@phd.unipi.it (S.B.); federica.chiellini@unipi.it (F.C.); lorenzo.biancalana@unipi.it (L.B.); guido.pampaloni@unipi.it (G.P.); 2 Institut des Sciences et Ingénierie Chimiques, Ecole Polytechnique Fédérale de Lausanne (EPFL), CH-1015 Lausanne, Switzerland; lucinda.batchelor495@gmail.com (L.K.B.); paul.dyson@epfl.ch (P.J.D.); 3 Institut für Chemie und Biochemie, Fabeckstr 34-36, 14195 Berlin, Germany; eferretti@zedat.fu-berlin.de (E.F.); Biprajit.Sarkar@fu-berlin.de (B.S.); 4 Dipartimento di Scienze Molecolari e Nanosistemi, Ca’ Foscari Università di Venezia, Via Torino 155, I-30170 Mestre (VE), Italy; markos@unive.it; 5 Dipartimento di Chimica Industriale “Toso Montanari”, Università di Bologna, Viale Risorgimento 4, I-40136 Bologna, Italy; stefano.zacchini@unibo.it

**Keywords:** bioorganometallic chemistry, metal-based drugs, diiron complexes, vinyliminium ligand, sulphur, selenium

## Abstract

A series of diiron/tetrairon compounds containing a S- or a Se-function (**2a**–**d**, **4a**–**d**, **5a**–**b**, **6**), and the monoiron [FeCp(CO){SeC^1^(NMe_2_)C^2^HC^3^(Me)}] (**3**) were prepared from the diiron μ-vinyliminium precursors [Fe_2_Cp_2_(CO)( μ-CO){μ-η^1^: η^3^-C^3^(R’)C^2^HC^1^N(Me)(R)}]CF_3_SO_3_ (R = R’ = Me, **1a**; R = 2,6-C_6_H_3_Me_2_ = Xyl, R’ = Ph, **1b**; R = Xyl, R’ = CH_2_OH, **1c**), via treatment with S_8_ or gray selenium. The new compounds were characterized by elemental analysis, IR and multinuclear NMR spectroscopy, and structural aspects were further elucidated by DFT calculations. The unprecedented metallacyclic structure of **3** was ascertained by single crystal X-ray diffraction. The air-stable compounds (**3**, **4a**–**d**, **5a**–**b**, **6**) display fair to good stability in aqueous media, and thus were assessed for their cytotoxic activity towards A2780, A2780cisR, and HEK-293 cell lines. Cyclic voltammetry, ROS production and NADH oxidation studies were carried out on selected compounds to give insights into their mode of action.

## 1. Introduction

The serendipitous discovery of the anticancer properties of cisplatin led to a paradigm shift in the clinical treatment of cancer. Although cisplatin and second generation platinum-based drugs are efficacious against many types of cancer [1,2,3,4], their use is associated with some restrictions, such as a limited selectivity leading to adverse side-effects and intrinsic or acquired resistance [5,6,7,8,9]. These limitations have fueled the research for the development of anticancer agents based on transition metals other than platinum [10,11,12,13,14,15,16,17,18,19,20,21], and in this respect mono-iron cyclopentadienyl compounds have been investigated, with substituted ferrocenes emerging as highly promising candidates [22,23,24]. Nevertheless, studies on poly-iron organometallic complexes still remain rare [25], and also iron-carbonyl compounds have been scarcely explored in the field so far [26,27,28].

Sulphur and selenium are found in a variety of organic molecules with therapeutic properties [29,30], and organo-selenium compounds have especially aroused interest for their anticancer potential, exerting their action alone or in combination with other drugs [31,32,33,34]. In this regard, the synthetic conjugation of a selenium moiety with the IrCp* frame (Cp* = η^5^-C_5_Me_5_) was previously found to result in a high cytotoxicity against A2780 cancer cells [35]. Being relevant to key redox processes in living organisms, disulphide and diselenide functions, when incorporated within a drug structure, have been demonstrated to induce antiproliferative and apoptotic effects [36,37,38].

**Scheme 1:**
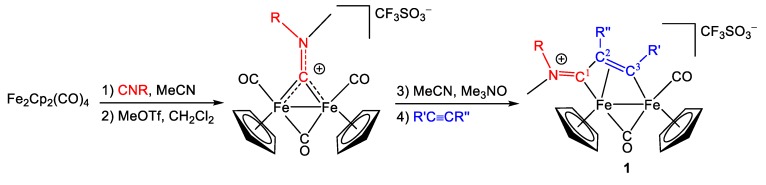
Synthesis of diiron complexes containing a bridging vinyliminium ligand, **1**, obtained by isocyanide (red) / alkyne (blue) coupling.

Recently, we reported on the antiproliferative behavior of diiron complexes comprising a bridging vinyliminium ligand, **1** [39], obtained via the sequential assembly of an isocyanide and an alkyne on Fe_2_Cp_2_(CO)_4_ (Scheme 1, Cp = η^5^-C_5_H_5_) [40,41,42]. Type **1** compounds possess some drug-like characteristics, i.e., they are based on a substantially nontoxic metal, they may be prepared on a multigram scale from cost effective precursors, they are stable in water media, and their solubility/lipophilicity can be regulated by an appropriate choice of ligand substituents. Preliminary experiments suggest that their cytotoxicity is mainly attributable to ROS generation triggered by either a single-electron reduction or slow compound fragmentation in aqueous media [39].

Former findings indicate that the vinyliminium ligand in type **1** compounds displays a versatile and rich chemistry, offering much scope for derivatization [43,44,45]. Herein, we will describe the synthesis and the characterization of a series of S- and Se-functionalized derivatives [42,43,44,45,46,47]. Cytotoxicity data concerning both cancer and non-cancer cell lines and experiments aimed to clarify the mechanism of action of the compounds are described.

## 2. Results and Discussion

### 2.1. Synthesis and Characterization of Compounds, and DFT Analysis

#### Synthesis and Characterization of Compounds

**Scheme 2:**
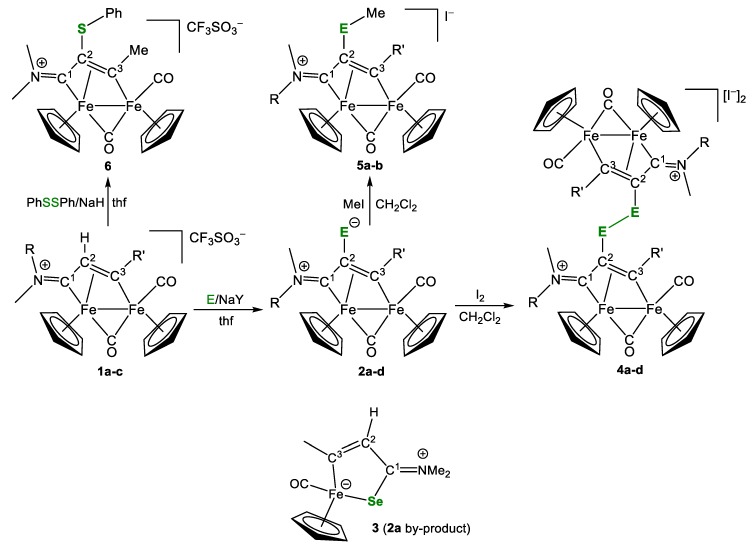
Synthesis of functionalized mono-, di- and tetrairon complexes via reactions of diiron vinyliminium compounds with elemental sulphur/selenium (E) and PhSSPh. **1a**: R = R’ = Me; **1b**: R = Xyl, R’ = Ph; **1c**: R = Xyl, R’ = CH_2_OH. **2a**: R = R’ = Me, E = Se; **2b**: R = Xyl, R’ = Ph, E = S, Y = H; **2c**: R = Xyl, R’ = CH_2_OH, E = S; **2d**: R = Xyl, R’ = Ph, E = Se, Y = OMe. **4a**: R = R’ = Me, E = Se; **4b**: R = Xyl, R’ = Ph, E = S; **4c**: R = Xyl, R’ = CH_2_OH, E = S; **4d**: R = Xyl, R’ = Ph, E = Se. **5a**: R = R’ = Me, E = Se; **5b**: R = Xyl, R’ = Ph, E = S. Xyl = 2,6-C_6_H_3_Me_2_.

Compounds **2a** and **2c** [48], **4c** [49] and **6** [50] were previously reported, whereas **2b**, **2d**, **3**, **4a**, **4b**, **4d**, **5a**, **5b**, and **6** are novel (Scheme 2). Once isolated, **2a**–**d** slowly decompose in contact with air, whereas **3**–**6** resulted indefinitely air-stable. The sodium hydride(methoxide)-promoted dehydrogenative chalcogenylation of **1a**–**c**, as described previously [48], provides access to the zwitterionic complexes **2a**–**d**, in 60%-80% yields. This formal [C^2^H]^+^/C^2^E substitution (E = S, Se) presumably proceeds through the initial single-electron reduction of the cationic part of **1a**–**c**. Consistent with this hypothesis, the monoiron complex **3**, maintaining the C^2^-H unit, is a side product of the reaction leading to **2a**, and may be viewed as the result of selenium incorporation along a fragmentation process initiated by electron transfer to **1a** [51]. The chalcogenido moiety in **2a**–**d** is readily oxidized with I_2_ to the dimeric iodide salts **4a**–**d**, containing an E-E bridge (77%–93%) [48]. Electrophilic methylation of **2a**–**d** affords **5a**–**b** (76%-86%). Instead, **6** is directly derived from **1a** (80%), trapping the [SPh] fragment along the reaction of **1a** with NaH [49].

According to combined X-ray diffraction analysis and NMR spectroscopy studies, the previously reported **2c** and related R = Xyl containing complexes exist both in solution and in the solid state in the Z form, i.e., displaying the bulky xylyl group far from the chalcogen atom [48]. The salient NMR spectroscopic features of the new compounds, **2b** and **2d**, are in good agreement with those of **2c** and analogues, thus indicating a Z configuration. For instance, the Cp rings and the methyl groups in the respective ^1^H NMR spectra are observed as follows: in **2b**, at 4.59, 4.58 (Cp), 3.70 (Me) and 2.65, 2.16 ppm (Xyl); in **2d**, at 4.62, 4.58 (Cp), 3.70 (Me) and 2.73, 2.16 ppm (Xyl); in [Fe_2_Cp_2_(CO)(μ-CO){μ-η^1^:η^3^-C(4-C_6_H_4_Me)C(S)CN(Me)(Xyl)}] [47], at 4.59, 4.55 (Cp), 3.69 (Me) and 2.65, 2.16 ppm (Xyl). The latter complex differs from **2b** in the presence of a 4-tolyl substituent in the place of Ph, and its structure was confirmed by X-ray diffraction.

**Figure 1:**
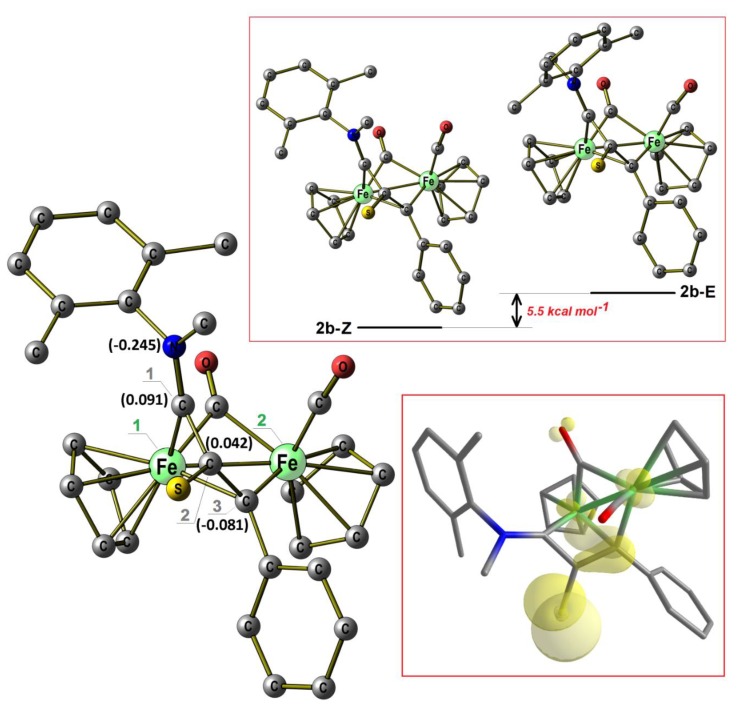
DFT-optimized structure of **2b**, Z isomer (C-PCM/ωB97X/def2-SVP calculation, chloroform as continuous medium). Hydrogen atoms are omitted for clarity. Selected computed bond lengths (Å): Fe(1)-Fe(2) 2.534; Fe(1)-C(μCO) 1.909; Fe(2)-C(μCO) 1.902; Fe(1)-C(3) 2.033; Fe(2)-C(3) 1.977; Fe(2)-C(2) 2.143; Fe(1)-C(1) 1.878; Fe(2)-C(CO) 1.769; Fe(1)-Cp(average) 2.082; Fe(2)-Cp(average) 2.105; C(3)-C(2) 1.424; C(2)-C(1) 1.437; C(2)-S 1.732; C(1)-N 1.299. Selected computed angles (°): Fe(1)-C(3)-C(2) 74.3; Fe(2)-C(3)-C(2) 123.2; C(1)-C(2)-C(3) 111.9; C(3)-C(2)-S 128.5; S-C(2)-C(1) 117.2; C(2)-C(1)-N 132.0. Selected Mulliken charges (a.u.) in parenthesis. Inset 1: Gibbs energy different between E and Z isomers of **2b** (EDF2/6-31G** calculations). Cartesian coordinates of the EDF2 geometries are collected in the SI. Inset 2: HOMO of **2b** (surface isovalue = 0.05 a.u.).

**Figure 2:**
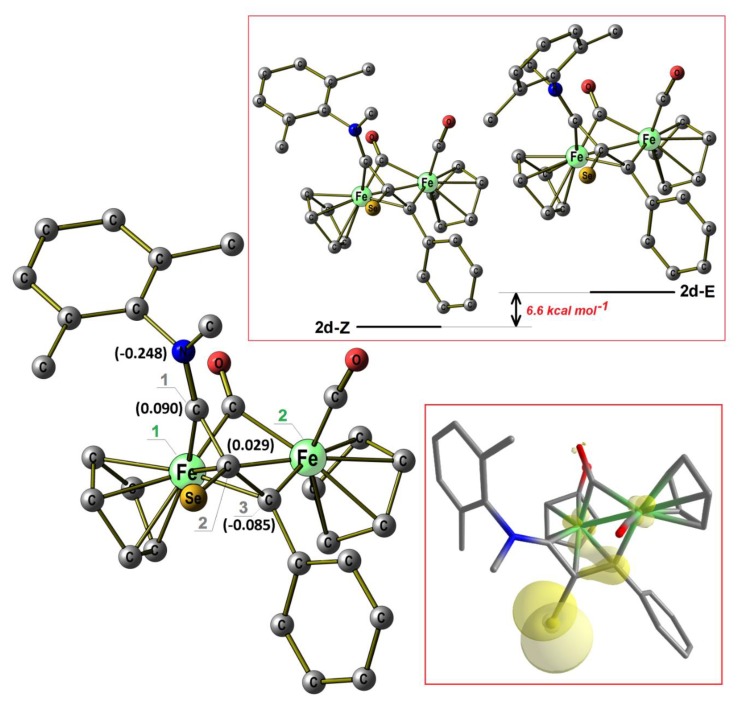
DFT-optimized structure of **2d**, Z isomer (C-PCM/ωB97X/def2-SVP calculation, chloroform as continuous medium). Hydrogen atoms are omitted for clarity. Selected computed bond lengths (Å): Fe(1)-Fe(2) 2.536; Fe(1)-C(μCO) 1.920; Fe(2)-C(μCO) 1.895; Fe(1)-C(3) 2.038; Fe(2)-C(3) 1.976; Fe(2)-C(2) 2.119; Fe(1)-C(1) 1.876; Fe(2)-C(CO) 1.770; Fe(1)-Cp(average) 2.082; Fe(2)-Cp(average) 2.104; C(3)-C(2) 1.419; C(2)-C(1) 1.430; C(2)-Se 1.896; C(1)-N 1.299. Selected computed angles (°): Fe(1)-C(3)-C(2) 73.1; Fe(2)-C(3)-C(2) 123.4; C(1)-C(2)-C(3) 113.0; C(3)-C(2)-Se 128.2; Se-C(2)-C(1) 116.7; C(2)-C(1)-N 133.0. Selected Mulliken charges (a.u.) in parenthesis. Inset1: Gibbs energy different between E and Z isomers of **2d** (EDF2/6-31G** calculations). Cartesian coordinates of the EDF2 geometries are collected in the SI. Inset2: HOMO of **2d** (surface isovalue = 0.05 a.u.).

DFT calculations confirm that the Z isomers of **2b** and **2d** are more stable than the E form by about 6 kcal mol^−1^ (Figure 1 and Figure 2). A comparison of computed bond lengths and angles indicates only small changes on replacing sulphur (**2b**) with selenium (**2d**). The most affected distance is Fe(2)-C(2), being 2.143 Å in **2b** and 2.119 Å in **2d** (CPCM/ωB97X calculations). The similarity between **2b** and **2d** is confirmed by the Mulliken population analysis, providing close values of partial charge for the μ-vinyliminium ligand in the two compounds. The higher stability of the Z isomers can be explained on the basis of the lower electrostatic repulsion between the chalcogen atom and the xylyl ring, as observable for instance in Figure S1 (Supporting information), where the electrostatic potential surfaces of *E*-**2b** and *Z*-**2b** are compared.

**Figure 3:**
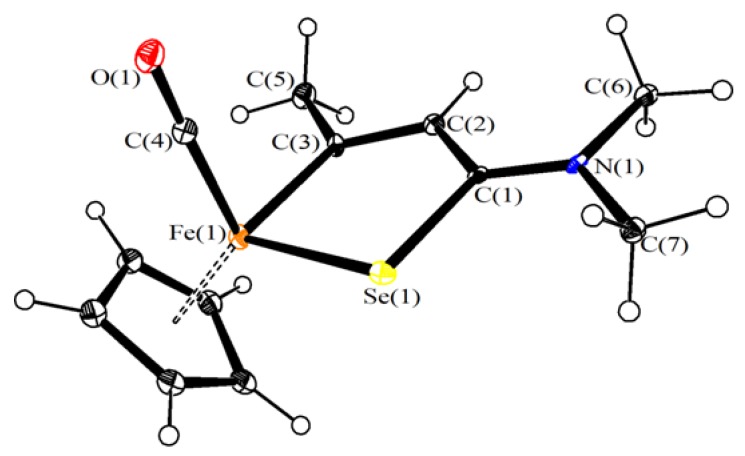
Molecular structure of [FeCp(CO){SeC^1^(NMe_2_)C^2^HC^3^(Me)}], **3**. Displacement ellipsoids are at the 30% probability level.

The structure of **3** was ascertained by single crystal X-ray diffraction (Figure 3, Table 1). Both C(1)-N(1) [1.28(6) and 1.27(6) Å for the two independent molecules present within the unit cell] and C(2)-C(3) [1.39(6) and 1.39(6) Å] distances show a significant double bond character, whereas C(1)-C(2) [1.46(6) and 1.43(6) Å] is essentially a C(sp^2^)-C(sp^2^) single bond with limited π-character. The Fe(1)-C(3) bond [1.96(4) and 1.92(5) Å] is elongated with respect to a pure terminal Fe^II^-alkylidene, revealing a vinyl character [52,53,54,55]. The Fe(1)-Se(1) distance [2.391(8) and 2.357(9) Å] is in keeping with previously reported iron(II)-selenide bonds [56,57,58,59,60]. The perfectly planar five-membered ring [mean deviation from the Fe(1)-Se(1)-C(1)-C(2)-C(3) least-squares plane 0.0094 and 0.0409 Å] can be described as a zwitterionic ferra-selenophene-iminium, and to the best of our knowledge is unprecedented in organometallic chemistry.

The ^1^H and ^13^C NMR spectra of 3 (acetone-*d*_6_ solution) display two resonances for the N-bound methyls, in accordance with the iminium description of the [C^1^-NMe_2_] moiety. Signals attributable to the C^1^, C^2^ and C^3^ carbons are observed at 218.6, 137.4 and 199.3 ppm, respectively, while the selenium centre is observed at 285.7 ppm in the ^77^Se NMR spectrum.

In both **2b** and **2d**, the HOMO is localized on a p-type orbital of E (E = S, Se) and to a lesser extent on C^3^, C^2^ and on the iron centers (see Figure 1 and Figure 2): this explains why the chalcogen atom E is the most probable site for molecular oxidation or electrophilic attack. The HOMO of **2d** is located 0.21 eV higher than in **2b**, so I_2_-oxidation of **2d** to **4d** is 6.5 kcal mol^−1^ more favorable than the analogous reaction for **2b**. Presumably, **4b** and **4d** containing xylyl groups, maintain the Z configuration of the N-substituents adopted in their precursors **2b** and **2d** [48]. Indeed, the NMR spectra of **4a**–**d** suggest the presence of a single species in solution. On going from **2d** to **4d**, the Se center undergoes significant deshielding in the ^77^Se NMR spectrum (from 282.5 to 556.4 ppm). DFT-optimized structures of **4b** and **4d** are shown in Figures S2 and S3.

The salient IR and NMR features of **5a**–**b** are typical of cationic vinyliminium complexes. In particular, the NMR spectra of **5b** closely resemble those reported for *Z*-[Fe_2_Cp_2_(CO)(μ-CO){μ-η^1^:η^3^-C(4-C_6_H_4_Me)C(SH)CN(Me)(Xyl)}]CF_3_SO_3_, **5c**, whose structure was confirmed by X-ray diffraction [e.g.,: δ(^1^H, **5b**/**5c**) = 5.16/4.99, 5.10/4.97 (Cp), 3.54/3.54 (NMe), 2.61/2.58, 2.07/2.04 (Xyl) ppm; δ(^13^C, **5b**/**5c**)/ppm = 227.9/227.8 (C^1^); 68.4/68.2 (C^2^); 205.6/208.5 (C^3^)] [39]. In the ^77^Se NMR spectrum of **5a** (acetone-d^6^ solution), the selenido unit gives rise to a signal at 187.4 ppm.

### 2.2. Electrochemistry

Compounds **4c** and **5a** were selected for electrochemical characterization in acetonitrile, which was extended to the respective precursors **1c** and **2c**, and also to **1a**. The main results are summarized in Table 2, and all cyclic voltammetric profiles (referred to the ferrocenium/ferrocene redox couple) are provided in the Supporting Information (Figures S4–S14). In general, the investigated complexes exhibit one electrochemical reduction process, which occurs reversibly for **1a** and **5a** on the time scale of the experiment, respectively at −1.37 V and −1.29 V. However, the reduction observed for **1a** is complicated by either two different processes occurring at very similar potentials, or a single process occurring in two steps (Figure S6). As a consequence, a slightly high peak-to-peak separation (∆E_p_) of 108 mV was recognized. Furthermore, **1a** displays an irreversible oxidation at +0.65 V, whereas in the case of **5a** several irreversible oxidation reactions were detected in the potential range from −0.44 V to +0.66 V.

As discussed above, the chalcogenido moiety of **2c** can be chemically oxidized to **4c** (Scheme 2), and the same conversion was investigated using electrochemical techniques. As expected, the cyclic voltammogram (CV) of **2c** shows an irreversible oxidation at +0.12 V, ascribable to the generation of the cationic part of **4c**. Correspondingly, the CV profile of **4c** shows an irreversible reduction at −0.78 V, that could be assigned to the formation of **2c** [61]. Further considerations are prevented due to the presence of iodide as the counteranion in **4c**, which is redox active and leads to the deposition of degradation products on the surface of the working electrode.

### 2.3. Cytotoxicity Studies and Stability in Aqueous Media

The air sensitive compounds **2a**–**d** were excluded from the biological tests. The remaining compounds were preliminarily evaluated for their stability in aqueous media (data summarized in Table 6). The ionic compounds **4a**–**d**, **5a**–**b** and **6**, which are slightly soluble in water, and **3** did not undergo significant modification in DMSO-*d*_6_/D_2_O solution after 72 h at ca. 37 °C, according to ^1^H NMR spectroscopy (see Experimental for details). Approximately 50% degradation of **4b** to unidentified species was detected after a further 72 h following addition of NaCl to the solution, whereas **4a,c,d** did not change under the same conditions. IR spectroscopy was used to estimate the stability of **4a**–**d**, **5a** and **6** in contact with cell culture medium at 37 °C. Compounds **4b**, **4d**, **5a**, and **6** remained intact after 72 h, whereas **4a** and **4c** were recovered at the end of the experiment together with other carbonyl species. Indeed, a significant amount of **2c** was detected to be produced from **4c**.

Compounds **3**–**6** were assessed for their cytotoxicity against cisplatin sensitive (A2780) and cisplatin resistant (A2780cisR) human ovarian carcinoma cells, and the non-tumoral human embryonic kidney (HEK-293) cell line (see Table 3 and Experimental for details). Cisplatin and [(η^6^-*p*-cymene)RuCl_2_(κ*P*-pta)] (RAPTA-C)[62] were evaluated as positive and negative controls, respectively.

Three tetrairon complexes, i.e., **4b**–**4d**, containing a S–S or a Se–Se bridge, and the diiron vinyliminium complexes **5b** and **6**, bearing a thioether function, possess potent cytotoxicity against the cancer cell lines, with IC_50_ values in the low micromolar/nanomolar range. In particular, the activity of **4b**, **4d**, and **5d** is superior than that of cisplatin and appears to overcome resistance issues, since comparable IC_50_ values were determined on the A2780 and A2780cisR cell lines. However, selectivity is not observed compared to the HEK-293 cell line, apart from a moderate selectivity shown by **5a**. The introduction of a Se–Se bridge on **1a** leads to a dramatic decrease in activity, the diselenide derivative **4a** being inactive towards all the investigated cell lines. In general, the strongest cytotoxic effect promoted by **4b**,**d**, compared to **4a**,**c**, reflects the higher stability in aqueous media of the former respect to the letter (see above).

### 2.4. ROS Production and NADH Oxidation

**Figure 4:**
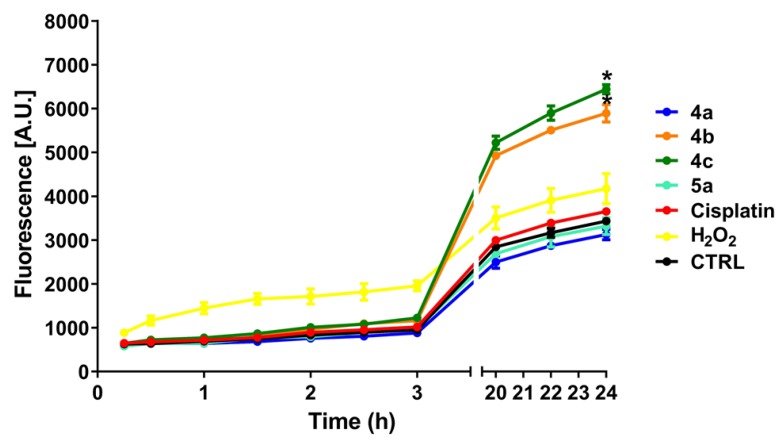
Fluorescence kinetics measurements of intracellular reactive oxygen species (ROS). A2780 cells incubated for 24 h with 10 µM of iron compounds at 37 °C.

We previously hypothesized that the cytotoxicity of diiron vinyliminium compounds, **1**, is mainly ascribable to redox mechanisms (see Introduction). As suggested by the DFT outcomes, electrochemical investigations and stability data (see above), the tetrairon-bis-cationic complexes **4a**–**d** are susceptible to relatively facile reduction due to feasible disulphide(diselenide) to sulphide(selenide) conversion. Even the reduction of the selenido-vinyliminium **5a** appears slightly more favorable with respect to analogous non-functionalized vinyliminium complexes (Table 2) [63]. Therefore, the cytotoxicity of the S- and Se-derivatives, and especially **4b**–**d**, is expected to involve interference of cellular redox processes. In order to investigate this aspect, we assessed the production of intracellular ROS levels induced by a selection of complexes (fluorescence measurements, using the peroxide-sensitive probe DCFH-DA). Thus, A2780 cells were continuously exposed to **4a**, **4b**, **4c**, **5a**, cisplatin (as a reference compound) and H_2_O_2_ (as a positive control). Treatment with **4b** and **4c** showed a significant increase in the level of ROS after ca. 20 h of treatment with respect to the positive control (Figure 4). Instead, **4a** and **5a** stimulated a ROS production close to that recorded for the basal levels; moreover, **4a** did not show a significant effect even at higher concentration (100 µM). The marked difference in behavior between **4a** (non cytotoxic) and **4b**–**d** indicates that the stimulation of ROS production could be indeed a privileged way of antiproliferative action for **4b**–**d**.

In order to further evaluate the ability of compounds to interfere with physiological redox processes, we determined the catalytic activity of **4a**, **4c**, **4d**, **5a**, and **6** in the aerobic oxidation of NADH, using a previously documented UV-Vis method (Table 4) [64]. Indeed, nicotinamide adenine dinucleotide (NAD^+^) and its reduced form (NADH) are important cofactors contributing to the maintenance of redox balance in cells [65], and the alteration of the NADH/NAD^+^ ratio has been implicated in the anticancer activity of various late transition metal complexes [64,66]. Cationic diiron vinyliminium compounds without chalcogen-functions, i.e., **1a** and **1c**, were also included in this study for comparison, together with FeSO_4_ as a reference compound. All tetrairon compounds **4a**, **4c**, **4d** displayed a moderate catalytic activity on NADH oxidation, comparable (or slightly superior) to that of their diiron precursors (**1a**, **1c**). Surprisingly, the diiron compounds **5a** and **6**, featuring selenoether/thioether moieties, were able to retard the oxidation of NADH with respect to the blank experiment. Notably, TONs at 25 h were significantly lower for **5a** and **6** than for FeSO_4_, the latter exhibiting no catalytic activity.

## 3. Experimental

### 3.1. Synthetic Procedures and Compound Characterization

*General details*. The preparation, purification and isolation of compounds were carried out under a N_2_ atmosphere using standard Schlenk techniques; once obtained, **3**–**6** were stored in air and **2a**–**d** were stored under N_2_. Solvents were purchased from Merck and distilled before use under N_2_ from appropriate drying agents. Organic reactants (TCI Europe or Merck) were commercial products of the highest purity available. Compounds **1a-e** [39,42], **2a,c** [48], **4c** [49], and **6** [50] were prepared according to published procedures. Chromatography separations were carried out on columns of deactivated alumina (Merck, 4% *w/w* water). Infrared spectra of solutions were recorded on a Perkin Elmer Spectrum 100 FT-IR spectrometer with a CaF_2_ liquid transmission cell (2300–1500 cm^−1^ range); IR spectra were processed with Spectragryph software [67]. NMR spectra were recorded at 298 K on a Bruker Avance II DRX400 instrument equipped with a BBFO broadband probe. Chemical shifts (expressed in parts per million) are referenced to the residual solvent peaks (^1^H, ^13^C) [68], or to external standard (^77^Se, SeMe_2_). ^1^H and ^13^C NMR spectra were assigned with the assistance of ^1^H-^13^C (*gs*-HSQC and *gs*-HMBC) correlation experiments [69]. Elemental analyses were performed on a Vario MICRO cube instrument (Elementar).

*Synthesis of [Fe_2_Cp_2_(CO)(μ-CO){μ-η^1^:η^3^-C^3^(Ph)C^2^(E)C^1^N(Me)(Xyl)}] (E* = *S*, **2b**; *E* = *Se*, **2d**).

Compound **2b** was prepared using the procedure reported in the literature for **2a** and **2c** [48], and a slight modification of the procedure was employed for **2d**.

**Chart 1:**
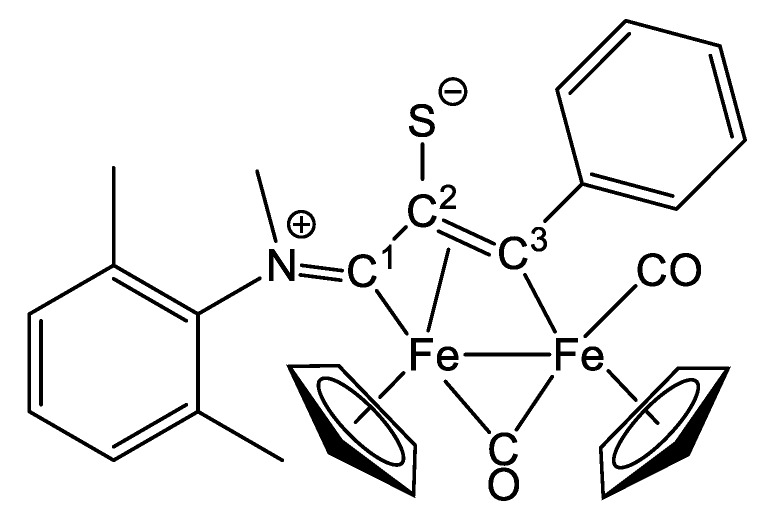
Structure of **2b**.

*[Fe_2_Cp_2_(CO)(μ-CO){μ-η^1^:η^3^-C^3^(Ph)C^2^(S)C^1^N(Me)(Xyl)}],* **2b** *(Chart 1).*

From **1b** (0.70 mmol), S_8_ (ca. 10 eq.) and NaH (4 eq.), see ref. [48]. Dark-green solid, yield 60%. Eluent for chromatography: CH_2_Cl_2_. Anal. calcd. for C_30_H_27_Fe_2_NO_2_S: C, 62.41; H, 4.71; N, 2.43; S, 5.56. Found: C, 63.06; H, 4.80; N, 2.40; S, 5.40. IR (CH_2_Cl_2_): ῦ/cm^−1^ = 1964vs (CO), 1791s (μ-CO), 1600m (C^1^N), 1581w (arom C-C). ^1^H NMR (CDCl_3_): δ/ppm = 7.68–7.28 (m, 8 H, C_6_H_5_ + C_6_*H*_3_Me_2_); 4.59, 4.58 (s, 10 H, Cp); 3.70 (s, 3 H, NMe); 2.65, 2.16 (s, 6 H, C_6_H_3_*Me*_2_). ^13^C{^1^H} NMR (CDCl_3_): δ/ppm = 264.2 (μ-CO); 235.4 (C^1^); 212.8 (CO); 195.7 (C^3^); 156.8 (*ipso*-C_6_H_5_); 142.3 (*ipso*-*C*_6_H_3_Me_2_); 135.7, 134.9, 129.3, 129.0, 127.8, 126.4 (C_6_H_5_ + *C*_6_H_3_Me_2_); 113.0 (C^2^); 90.6, 89.4 (Cp); 45.9 (NMe); 18.5, 18.0 (C_6_H_3_*Me*_2_). C^2^ observed via *g*-HSQC experiment.

**Chart 2:**
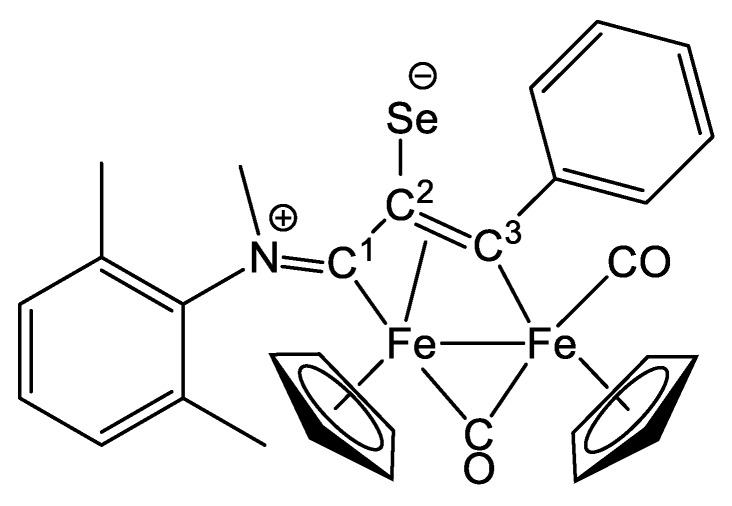
Structure of **2d**.

*[Fe_2_Cp_2_(CO)(μ-CO){μ-η^1^:η^3^-C^3^(Ph)C^2^(Se)C^1^N(Me)(Xyl)}]*, **2d***(Chart 2).*

A solution of **1b** (180 mg, 0.259 mmol) in THF (15 mL) was treated with gray Se (200 mg, 2.53 mmol) followed by NaOMe (35 mg, 0.648 mmol). The mixture was allowed to stir at room temperature for 1 h, then it was filtered through a short alumina pad, using neat THF as eluent, under protection from air. The filtrate was dried under vacuum. The resulting residue was dissolved in CH_2_Cl_2_ and the solution was charged on alumina. Elution with CH_2_Cl_2_ removed the impurities and a green band was collected using THF as eluent. The title compound was isolated as a brown solid upon removal of the solvent under vacuum. Yield 129 mg, 80%. Anal. calcd. for C_30_H_27_Fe_2_NO_2_Se: C, 57.73; H, 4.36; N, 2.24. Found: C, 57.61; H, 4.44; N, 2.18. IR (CH_2_Cl_2_): ῦ/cm^−1^ = 1967vs (CO), 1794s (μ-CO), 1604w (C^1^N), 1583w (arom C-C). ^1^H NMR (CDCl_3_): δ/ppm = 7.69–7.25 (m, 8 H, Ph + C_6_*H*_3_Me_2_); 4.62, 4.58 (s, 10 H, Cp); 3.70 (s, 3 H, NMe); 2.73, 2.16 (s, 6 H, C_6_H_3_*Me*_2_). ^13^C{^1^H} NMR (CDCl_3_): δ/ppm = 262.8 (μ-CO); 229.7 (C^1^); 212.4 (CO); 198.6 (C^3^); 157.7 (*ipso*-Ph); 141.9 (*ipso*-*C*_6_H_3_); 136.1, 135.0, 129.6, 129.1, 129.0, 128.8, 128.2, 128.0, 125.9, 125.3 (Ph + *C*_6_H_3_Me_2_); 90.7, 90.4 (Cp); 89.6 (C^2^); 47.0 (NMe); 18.6, 18.3 (C_6_H_3_*Me*_2_). ^77^Se NMR (CDCl_3_): δ/ppm = 282.5.

**Chart 3:**
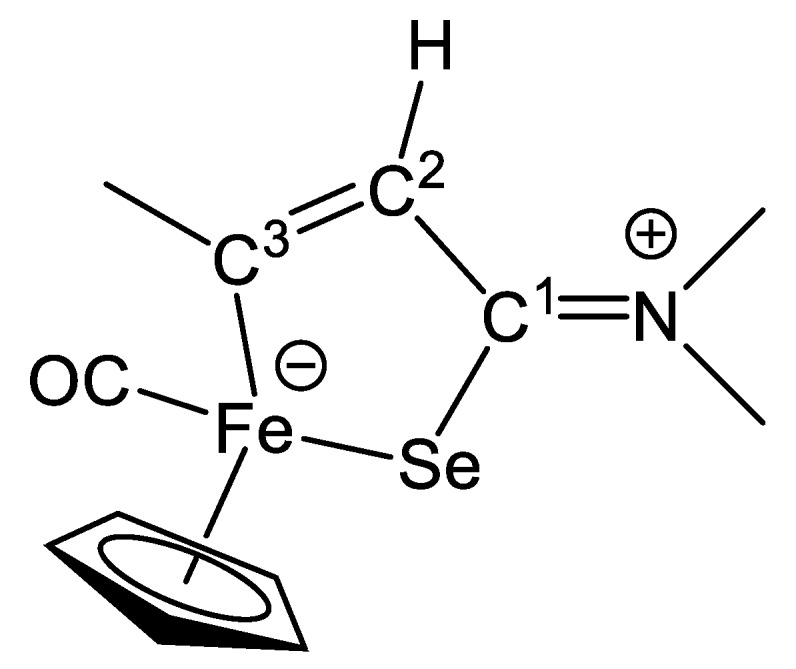
Structure of **3**.

*Synthesis of [FeCp(CO){SeC^1^(NMe_2_)C^2^HC^3^(Me)}]*, **3***(Chart 3)*.

The reaction mixture for the synthesis of **2a** was obtained as described in the literature, from **1a**, gray selenium and NaH [48]. This mixture was filtered through a short alumina pad using THF as eluent, then the volatiles were removed under vacuum. Subsequent alumina chromatography of the residue led to isolate a red fraction using neat diethyl ether as eluent, corresponding to **3**. Compound **3** was isolated as an air stable, red solid upon removal of the solvent under vacuum. Yield 16%. Anal. calcd. for C_12_H_15_FeNOSe: C, 44.48; H, 4.67; N, 4.32. Found: C, 44.12; H, 4.51; N, 4.39. IR (CH_2_Cl_2_): ῦ/cm^−1^ = 1921vs (CO), 1530m (C^3^=C^2^). ^1^H NMR (acetone-d_6_): δ/ppm = 7.36 (s, 1 H, C^2^-H); 4.59 (s, 5 H, Cp); 3.45, 3.28 (s, 6 H, NMe_2_); 2.77 (s, 3 H, C^3^-Me). ^13^C{^1^H} NMR (acetone-*d*_6_): δ/ppm = 252.3 (CO); 218.6 (C^1^); 199.3 (C^3^); 137.4 (C^2^); 82.3 (Cp); 44.8, 42.5 (NMe_2_); 40.1 (C^3^*Me*). ^77^Se NMR (acetone-*d*_6_): δ/ppm = 285.7. Crystals suitable for X-ray analysis were obtained from a diethyl ether solution layered with pentane and stored at −30 °C.

*Synthesis of [Fe_2_Cp_2_(CO)(μ-CO){μ-η^1^:η^3^-C^3^(R’)C^2^(E)C^1^N(Me)(R)}]_2_[I]_2_ (R = R’ = Me, E = Se,* **4a** *; R = Xyl, R’ = Ph, E = S,* **4b** *; R = Xyl, R’ = Ph, E = Se,* **4d** *).*

The title products were prepared using the procedure reported in the literature for **4c** [49]. 

**Chart 4:**
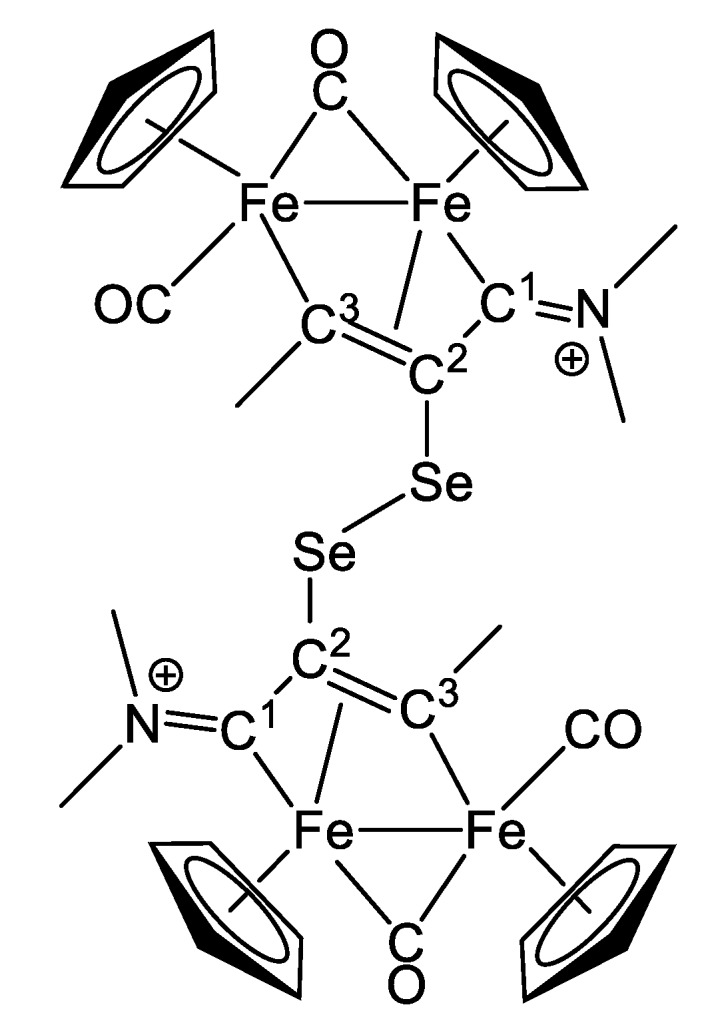
Structure of the cation of **4a**.

*[Fe_2_Cp_2_(CO)(μ-CO){μ-η^1^:η^3^-C^3^(Me)C^2^(Se)C^1^NMe_2_}]_2_[I]_2_*, **4a** (Chart 4).

From **2a** and I_2_, see ref. [49]. Red solid, yield 84%. Anal. calcd. for C_36_H_38_Fe_4_I_2_N_2_O_4_Se_2_: C, 36.10; H, 3.20; N, 2.34. Found: C, 36.05; H, 3.26; N, 2.41. IR (CH_2_Cl_2_): ῦ/cm^−1^ = 1995vs (CO), 1815s (μ-CO), 1671m (C^2^C^1^N). ^1^H NMR (acetone-d_6_): δ/ppm = 5.75, 5.34 (s, 20 H, Cp); 4.24, 4.12 (s, 12 H, NMe + C^3^Me); 3.62 (s, 6 H, NMe_2_). ^13^C{^1^H} NMR (acetone-d_6_): δ/ppm = 252.9 (μ-CO); 220.9 (C^1^); 210.0 (CO); 208.7 (C^3^); 92.1, 89.1 (Cp); 57.1 (C^2^); 51.6, 45.9 (NMe); 41.1 (C^3^*Me*). ^77^Se NMR (DMSO-d_6_): δ/ppm = 519.8.

**Chart 5:**
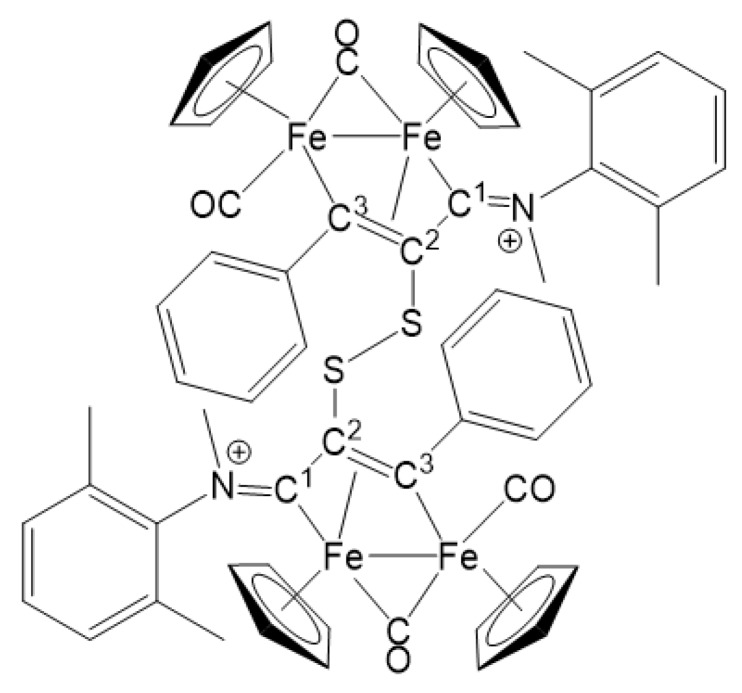
Structure of the cation of **4b**.

*[Fe_2_Cp_2_(CO)(μ-CO){μ-η^1^:η^3^-C^3^(Ph)C^2^(S)C^1^N(Me)(Xyl)}]_2_[I]_2_*, **4b***(Chart 5).*

From **2b** and I_2_, see ref. [49]. Dark-red solid, yield 77%. Anal. calcd. for C_60_H_54_Fe_4_I_2_N_2_O_4_S_2_: C, 51.17; H, 3.86; N, 1.99; S, 4.55. Found: C, 51.02; H, 3.94; N, 2.06; 4.69. IR (CH_2_Cl_2_): ῦ/cm^−1^ = 1994vs (CO), 1830s (μ-CO), 1611m (C^2^C^1^N), 1586w (arom C-C). ^1^H NMR (CD_2_Cl_2_): δ/ppm = 7.81–7.29 (m, 8 H, C_6_H_5_ + C_6_*H*_3_Me_2_); 5.10, 5.08 (s, 10 H, Cp); 3.21 (s, 3 H, NMe); 2.66, 2.10 (s, 6 H, C_6_H_3_*Me*_2_). ^13^C{^1^H} NMR (CD_2_Cl_2_): δ/ppm = 249.9 (μ-CO); 227.3 (C^1^); 210.1, 209.2 (CO + C^3^); 152.7 (*ipso*-C_6_H_5_); 140.1 (*ipso*-*C*_6_H_3_Me_2_); 134.3-126.7 (C_6_H_5_ + *C*_6_H_3_Me_2_); 90.6, 89.4 (Cp); 65.8 (C^2^); 45.9 (NMe); 18.8, 18.1 (C_6_H_3_*Me*_2_). *NMe* overlapped with solvent signal.

**Chart 6:**
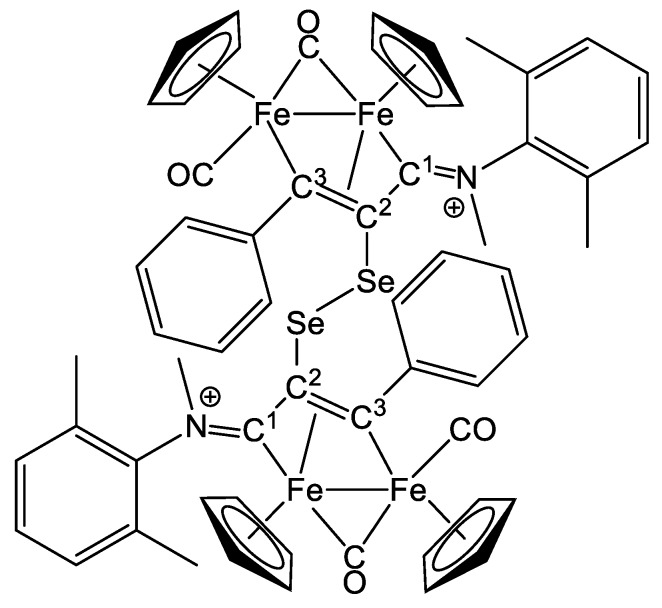
Structure of the cation of **4d**.

*[Fe_2_Cp_2_(CO)(μ-CO){μ-η^1^:η^3^-C^3^(Ph)C^2^(Se)C^1^N(Me)(Xyl)}]_2_[I]_2_*, **4d***(Chart 6).*

From **2b** and I_2_, see ref. [49]. Brown solid, yield 93%. Anal. calcd. for C_60_H_54_Fe_4_I_2_N_2_O_4_Se_2_: C, 47.97; H, 3.62; N, 1.86. Found: C, 47.85; H, 3.68; N, 1.93. IR (CH_2_Cl_2_): ῦ/cm^−1^ = 1994vs (CO), 1825s (μ-CO), 1616m (C^2^C^1^N), 1586w (arom C-C). ^1^H NMR (CD_3_CN): δ/ppm = 7.85–7.45, 7.36, 7.01 (m, 8 H, C_6_H_5_ + C_6_*H*_3_Me_2_); 5.04, 4.98 (s, 10 H, Cp); 3.49 (s, 3 H, NMe); 2.52, 2.14 (s, 6 H, C_6_H_3_*Me*_2_). ^77^Se NMR (DMSO-d_6_): δ/ppm = 556.4.

*Synthesis of [Fe_2_Cp_2_(CO)(μ-CO){μ-η^1^:η^3^-C^3^(R’)C^2^(EMe)C^1^N(Me)(R)}]I (R = R’ = Me, E = Se,* **5a** *; R = Xyl, R’ = Ph, E = S,* **5b** *).*

*General procedure*. Compound **2a**–**b** (ca. 0.50 mmol) was dissolved in CH_2_Cl_2_ (15 mL), and MeI (1.5 equivalents) was added to the solution. The resulting mixture was stirred at room temperature for 2 h, and then charged on an alumina column. Elution with CH_2_Cl_2_ allowed to separate impurities, then the fraction corresponding to the product was collected using MeCN/MeOH (95/5 *v*/*v*) as eluent.

**Chart 7:**
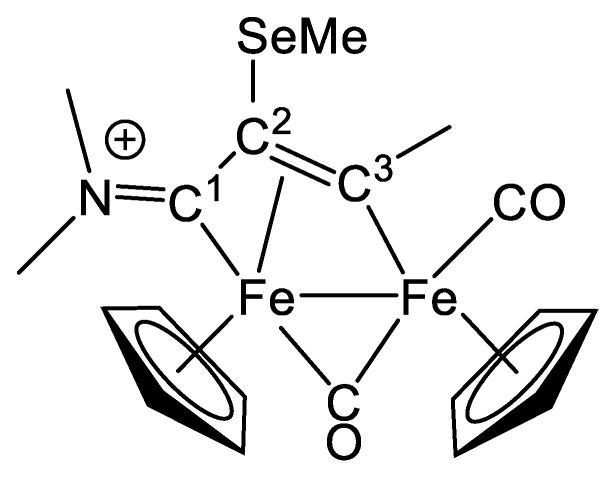
Structure of the cation of **5a**.

*[Fe_2_Cp_2_(CO)(μ-CO){μ-η^1^:η^3^-C^3^(Me)C^2^(SeMe)C^1^NMe_2_)}]I*, **5a***(Chart 7).*

From **2a** and MeI. Brown solid, yield 86%. Eluent for chromatography: MeOH. Anal. calcd. for C_27_H_31_Fe_2_INO_2_Se: C, 45.10; H, 4.35; N, 1.95. Found: C, 44.90; H, 4.27; N, 1.98. IR (CH_2_Cl_2_): ῦ/cm^−1^ = 1992vs (CO), 1813s (μ-CO), 1667m (C^2^C^1^N). ^1^H NMR (acetone-*d*_6_): δ/ppm = 5.59, 5.24 (s, 10 H, Cp); 4.13 (s, 3 H, C^3^Me); 4.03, 3.32 (s, 6 H, NMe_2_); 2.35 (s, 3 H, SeMe). ^13^C{^1^H} NMR (acetone-*d*_6_): δ/ppm = 255.4 (μ-CO); 220.3 (C^1^); 210.3 (CO); 205.0 (C^3^); 91.3, 88.9 (Cp); 65.1 (C^2^); 47.6, 44.9 (NMe); 39.1 (C^3^*Me*); 6.7 (SeMe). ^77^Se NMR (DMSO-*d*_6_): δ/ppm = 187.4.

**Chart 8:**
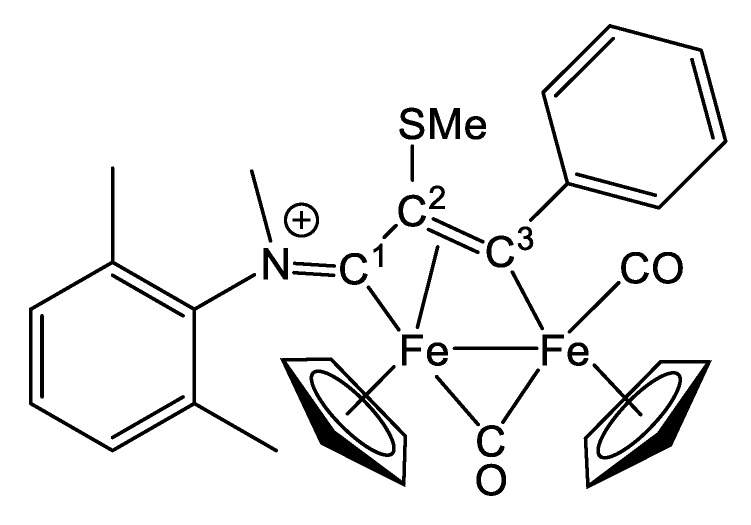
Structure of the cation of **5b**.

*[Fe_2_Cp_2_(CO)(μ-CO){μ-η^1^:η^3^-C^3^(Ph)C^2^(SMe)C^1^N(Me)(Xyl)}]I*, **5b***(Chart 8)*.

From **2b** and MeI. Dark-brown solid, yield 76%. Eluent for chromatography: THF. Anal. calcd. for C_31_H_30_Fe_2_INO_2_S: C, 51.77; H, 4.20; N, 1.95; S, 4.46. Found: C, 51.65; H, 4.26; N, 2.03; S, 4.40. IR (CH_2_Cl_2_): ῦ/cm^−1^ = 1993vs (CO), 1829s (μ-CO), 1611m (C^2^C^1^N), 1587w (arom C-C). ^1^H NMR (CDCl_3_): δ/ppm = 7.91, 7.75, 7.54–7.41, 6.98 (m, 8 H, C_6_H_5_ + C_6_*H*_3_Me_2_); 5.16, 5.10 (s, 10 H, Cp); 3.54 (s, 3 H, NMe); 2.61, 2.07 (s, 6 H, C_6_H_3_*Me*_2_); 2.12 (s, 3 H, SMe). ^13^C{^1^H} NMR (CDCl_3_): δ/ppm = 250.7 (μ-CO); 227.9 (C^1^); 210.7 (CO); 205.6 (C^3^); 152.5 (*ipso*-C_6_H_5_); 140.5 (*ipso*-*C*_6_H_3_Me_2_); 135.8, 134.2, 134.1, 130.2, 130.0, 129.2, 128.1, 127.4, 127.3, 127.1, 125.5 (C_6_H_5_ + *C*_6_H_3_Me_2_); 93.3, 88.8 (Cp); 68.4 (C^2^); 51.5 (NMe); 19.6 (SMe); 18.2, 18.0 (C_6_H_3_*Me*_2_).

### 3.2. X-Ray Crystallography

Crystal data and collection details for **3** are reported in Table 5. Data were recorded on a Bruker APEX II diffractometer equipped with a PHOTON100 detector using Mo–Kα radiation. The crystal appeared to be non-merohedrally twinned. The program CELL_NOW (G. M. Sheldrick, *CELL_NOW*, Version 2008/4, 2008) was used in order to determine the two twin domains and their orientation matrices. After integration, data were corrected for Lorentz polarization and absorption effects (empirical absorption correction TWINABS) [70]. The structure was solved by direct methods and refined by full-matrix least-squares based on all data using *F*^2^ [71]. Hydrogen atoms were fixed at calculated positions and refined using a riding model. All non-hydrogen atoms were refined with anisotropic displacement parameters. Refinement was performed using the instruction HKLF 5 in SHELXL and one BASF parameter, which refined as 0.276(7). Because of the twinning, several restraints were applied during refinement. All the atoms were restrained to have similar thermal parameters (SIMU line in SHLEXL, s.u. 0.01). All C, O, and N atoms were restrained to isotropic like behavior (ISOR line in SHELXL, s.u. 0.01).

### 3.3. Computational Studies

The electronic structures of the compounds were optimized using the range-separated ωB97X DFT functional [72,73,74] in combination with Ahlrichs’ split-valence-polarized basis set [75]. The C-PCM implicit solvation model was added to ωB97X calculations, considering chloroform as a continuous medium [76,77].

Preliminary optimizations were carried out using the hybrid-GGA EDF2 functional [78] in combination with the 6-31G(d,p) basis set [79]. The stationary points were characterized by IR simulations (harmonic approximation), from which zero-point vibrational energies and thermal corrections (T = 25 °C) were obtained. Simulated IR spectra were used to assign the experimentally observed signals [80]. The software used were Gaussian 09 (Gaussian, Inc: Wallingford, CT, USA) [81] and Spartan ‘16 [82]. Cartesian coordinates of the DFT-optimized structures are collected in a separated .xyz file.

### 3.4. Stability in Aqueous Solutions

Each compound (ca. 10 mg; **3**, **4a**–**d**, **5a**–**b**, **6**) was dissolved in DMSO-*d*_6_ (0.4 mL), then the solution was diluted with variable volumes of D_2_O. The resulting solution was kept at 37 °C for 72 h. Subsequent ^1^H NMR spectra revealed the presence of the respective starting materials together with minor decomposition products (<10%). NaCl was added in ca. 0.05 M concentration to the solutions containing **4a**–**d**, and the obtained mixtures were kept at 37 °C for 72 h before ^1^H NMR spectra were recorded (Table 6). In order to perform tests in contact with a cell culture medium, compounds **4c**–**d**, **5a** and **6** (ca. 4 mg) were dissolved in DMSO (ca. 1 mL) in a glass tube, then RPMI-1640 medium with sodium bicarbonate, without l-glutamine and phenol red (ca. 3 mL, Merck), was added. The resulting mixture was maintained at 37 °C for 72 h, then it was allowed to cool to room temperature. Dichloromethane (ca. 4 mL) was added, and the mixture was vigorously shaken. An aliquot of the organic phase was analyzed by IR spectroscopy (Table 6).

### 3.5. Electrochemistry

Cyclic voltammograms were measured under an atmosphere of argon using standard Schlenk techniques with a Palmsens4 potentiostat by working with anhydrous and degassed solutions of acetonitrile (MeCN). MeCN was dried and distilled under Ar from the appropriate drying agent (CaH_2_), and thoroughly deoxygenated with Ar prior to use. The samples were measured at a concentration of 0.1 M and using 0.1 M NBu_4_PF_6_ (Merck) as conductive salt. A glassy carbon electrode was used as working electrode, a coiled platinum wire as counter electrode, and a silver wire as a pseudo-reference electrode. Ferrocene (or decamethylferrocene) was added as an internal standard and all spectra were referenced to the ferrocenium/ferrocene couple (Fc^+^/Fc).

### 3.6. Cell Culture and Cytotoxicity Studies

Human ovarian carcinoma (A2780 and A2780cisR) cell lines were obtained from the European Collection of Cell Cultures. The human embryonic kidney (HEK-293) cell line was obtained from ATCC (Merck, Buchs, Switzerland). Penicillin streptomycin, RPMI 1640 GlutaMAX (where RPMI = Roswell Park Memorial Institute), and DMEM GlutaMAX media (where DMEM = Dulbecco’s modified Eagle medium) were obtained from Life Technologies, and fetal bovine serum (FBS) was obtained from Merck. The cells were cultured in RPMI 1640 GlutaMAX (A2780 and A2780cisR) and DMEM GlutaMAX (HEK-293) media containing 10% heat-inactivated FBS and 1% penicillin streptomycin at 37 °C and CO_2_ (5%). The A2780cisR cell line was routinely treated with cisplatin (2 μM) in the media to maintain cisplatin resistance. The cytotoxicity was determined using the 3-(4,5-dimethyl 2-thiazolyl)-2,5-diphenyl-2*H*-tetrazolium bromide (MTT) assay [83]. Cells were seeded in flat-bottomed 96-well plates as a suspension in a prepared medium (100 μL aliquots and approximately 4300 cells/well) and preincubated for 24 h. Stock solutions of compounds were prepared in DMSO and were diluted in medium. The solutions were sequentially diluted to give a final DMSO concentration of 0.5% and a final compound concentration range (0–200 μM). Cisplatin and RAPTA-C [62] were tested in aqueous solution as a positive (0–100 μM) and negative (200 μM) controls, respectively. The compounds were added to the preincubated 96-well plates in 100 μL aliquots, and the plates were incubated for a further 72 h. MTT (20 μL, 5 mg/mL in Dulbecco’s phosphate buffered saline) was added to the cells, and the plates were incubated for a further 4 h. The culture medium was aspirated and the purple formazan crystals, formed by the mitochondrial dehydrogenase activity of vital cells, were dissolved in DMSO (100 μL/well). The absorbance of the resulting solutions, directly proportional to the number of surviving cells, was quantified at 590 nm using a SpectroMax M5e multimode microplate reader (using SoftMax Pro software, version 6.2.2). The percentage of surviving cells was calculated from the absorbance of wells corresponding to the untreated control cells. The reported IC_50_ values are based on the means from two independent experiments, each comprising four tests per concentration level.

### 3.7. ROS Production Assessment

The intracellular increase of reactive oxygen species (ROS) upon treatment with the analyzed complexes was measured by using the DCFH-DA (2′,7′-dichlorodihydrofluorescein diacetate, Merck) assay, based on cellular uptake of the non-fluorescent diacetate following deacetylation by esterases (2′,7′-dichlorodihydrofluorescein, DCFH) and oxidation to the fluorescent dichlorofluorescein (2′,7′-dichloro-fluorescein, DCF) [84]. A2780 cells were seeded at concentration of 4300 cells/well/90 µL of complete growth medium into 96-well plates and allowed to proliferate for 24 h. Then cells were treated following the manufacturer’s protocol. Briefly, the culture medium was supplemented with 100 µL of the fluorogenic probe solution and cells were incubated under standard tissue culture conditions of 5% CO_2_ at 37 °C. After 1 h, the cells were exposed with a final concentration of 10 µM of the tested compounds and maintained at 5% CO_2_ at 37 °C; H_2_O_2_ 100 µM was used as a positive control. Stock solutions of compounds were prepared as described above. Cells incubated with DMSO at a concentration of 0.1% in supplemented RPMI were used as control. The fluorescence was measured over 24 h with an excitation wavelength of 485 nm and with a 535 nm emission filter by Multilabel Counter (PerkinElmer, Waltham, USA). Analysis was conducted in triplicate and experimental data were reported as mean ± SD. Statistical differences were analyzed using one-way analysis of variance (ANOVA) and a Tukey test was used for post hoc analysis. A *p*-value <0.05 was considered as statistically significant.

### 3.8. Catalytic NADH Oxidation

NADH was stored at –20 °C under N_2_; a stock NADH solution (2.3 × 10^−4^ mol L^−1^) was prepared in phosphate buffered aqueous solution (Na_2_HPO_4_/NaH_2_PO_4_; 5.5 × 10^−3^ mol∙L^−1^, pH = 7.2) and stored at 4 °C. Stock solutions of iron compounds (**1a**, **4a**,**c**,**d**, **5a**, **6**; 2.0 × 10^−4^ mol∙L^−1^) were prepared in DMSO immediately before use. FeSO_4_ was used as a reference compounds (stock solution prepared in H_2_O). Solutions of each iron compound (0.35 mL) and NADH (6.6 mL) were mixed, resulting in a 5% DMSO aqueous solution containing 2.2 × 10^−4^ M NADH and 1.0∙× 10^−5^ M iron compound (4.5% mol). The solution was stirred at 37 °C for 25 h and periodically analyzed by UV-Vis spectroscopy (260–460 nm) using PMMA cuvettes (1.0 cm path-length). Turnover numbers were calculated as TON = c(0)/c_Fe_∙[A(0) − A(t)]/A(0) where A is the absorbance at λ_max_ = 339 nm; c(0) and c_Fe_ are the initial molar concentrations of NADH and the selected Fe compound, respectively (Table 4).

## 4. Conclusions

The bridging vinyliminium ligand in cationic diiron complexes can be modified by introducing sulphur- or selenium-functions according to well defined regio- and stereoselective reaction pathways. Some of the resulting, air stable diiron and tetrairon compounds display a strong antiproliferative activity against human ovarian carcinoma cell lines, the activity of some compounds on the A2780 cell line being superior than that of cisplatin and substantially maintained on the A2780cisR resistant cell line. Experiments suggest that the chalcogen function (especially the presence of an E–E bridge) is associated with good stability in aqueous media, enhancing interference of compounds with cellular redox processes.

## Figures and Tables

**Table 1:** Selected bond lengths (Å) and angles (°) for **3**.

	Molecule 1	Molecule 2
Fe(1)-Se(1)	2.391(8)	2.357(9)
Fe(1)-Cp	2.07(5)–2.17(5)	2.09(5)–2.10(5)
Fe(1)-C(4)	1.74(4)	1.83(4)
Fe(1)-C(3)	1.96(4)	1.92(5)
C(4)-O(1)	1.12(6)	1.09(5)
Se(1)-C(1)	1.90(4)	1.88(5)
C(1)-C(2)	1.46(6)	1.43(6)
C(2)-C(3)	1.39(6)	1.39(6)
C(3)-C(5)	1.50(6)	1.43(7)
C(1)-N(1)	1.28(6)	1.27(6)
N(1)-C(6)	1.53(6)	1.50(6)
N(1)-C(7)	1.52(5)	1.52(6)
Se(1)-Fe(1)-C(3)	84.9(13)	86.6(14)
Fe(1)-C(4)-O(1)	178(4)	171(4)
Fe(1)-Se(1)-C(1)	96.5(13)	98.3(14)
Se(1)-C(1)-C(2)	115(3)	108(3)
C(1)-C(2)-C(3)	118(4)	127(4)
C(2)-C(3)-Fe(1)	126(3)	119(4)
Sum at N(1)	360(6)	360(6)
Sum at C(1)	360(6)	359(6)
Sum at C(3)	360(6)	360(6)

**Table 2:** Overview of the main oxidation and reduction potentials (V vs. Fc^+^/Fc) at a scan rate of 100 mV/s determined by cyclic voltammetry in MeCN for selected iron complexes. The peak-to-peak separation (ΔE_p_) is determined by the difference between two peak potentials for a given redox couple. ^a^E_pa_ for an irreversible process.

Compound	Oxidation [V]	Reduction [V]	ΔE_p_ (red) [mV]
**1a**	+0.65 ^a^	−1.37	108
**1c**	+0.73 ^a^	−1.35 ^a^	-
**2c**	+0.12 ^a^	−1.7 ^a^	-
**4c**	-	−0.78 ^a^	-
**5a**	−0.44 ^a^ ÷ +0.66 ^a^	−1.29	87

**Table 3:** IC_50_ values (μM) determined for compounds **3**, **4a**–**d** (and their vinyliminium precursors **1a**–**c**), **5a**–**b**, **6**, cisplatin and RAPTA-C on human ovarian carcinoma (A2780), human ovarian carcinoma cisplatin resistant (A2780CisR) and human embryonic kidney (HEK-293) cell lines after 72 h exposure. Values are given as the mean ± SD. ^a^ See reference [39].

Compnd.	A2780	A2780cisR	HEK-293
**1a** ^a^	35 ± 3	86 ± 7	>200
**1b** ^a^	0.50 ± 0.06	1.2 ± 0.2	2.4 ± 0.2
**1c** ^a^	11.6 ± 0.6	21.2 ± 1.6	13.4 ± 1.0
**3**	16.1 ± 1.3	20 ± 2	19 ± 2
**4a**	>200	>200	>200
**4b**	0.6 ± 0.1	1.2 ± 0.6	0.72 ± 0.04
**4c**	5.7 ± 0.8	12.8 ± 0.7	9.1 ± 0.7
**4d**	1.4 ± 0.2	2.8 ± 0.3	2.2 ± 0.6
**5a**	15.6 ± 0.8	28 ± 2	26 ± 3
**5b**	0.5 ± 0.2	1.4 ± 0.2	0.7 ± 0.1
**6**	3.7 ± 0.4	14 ± 2	6.7 ± 0.6
cisplatin	2.7 ± 0.1	26 ± 3	10.0 ± 0.7
RAPTA-C	>200	>200	>200

**Table 4:** Turnover numbers (TON) of iron compounds (10 μM) in the aerobic oxidation of NADH (220 μM) in a 5% DMSO phosphate buffered solution at 37 °C after 25 h. FeSO_4_ used as a reference compound.

Compound	TON
**4a**	3.8
**4c**	4.1
**4d**	3.7
**5a**	1.8
**6**	1.6
**1a**	3.7
**1c**	3.5
FeSO_4_^[a]^	2.3

[a] NADH oxidation over time not significantly different from the blank experiment.

**Table 5:** Crystal data and measurement details for **3**.

	3
Formula	C_12_H_15_FeNOSe
FW	324.06
T, K	100(2)
*λ*, Å	0.71073
Crystal system	Monoclinic
Space group	*P*c
*a*, Å	13.454(3)
*b,* Å	7.675(2)
*c*, Å	12.285(2)
*β*,°	99.07(3)
Cell Volume, Å^3^	1252.5(5)
Z	4
*D_c_*, g∙cm^−3^	1.719
μ, mm^−^^1^	4.088
F(000)	648
Crystal size, mm	0.21 × 0.19 × 0.15
θ limits,°	1.533–24.999
Reflections collected	11149
Independent reflections	2119 [*R_int_* = 0.0687]
Data/restraints/parameters	2119/350/290
Goodness on fit on F^2^	1.116
*R*_1_ (*I* > 2σ(*I*))	0.1175
*wR*_2_ (all data)	0.2914
Largest diff. peak and hole, e Å^−3^	1.622/–1.857

**Table 6:** Overview of stability of compounds in aqueous media.

Comp.	Stability in DMSO-*d*_6_/D_2_O (*v*/*v*) + NaCl (0.05 M) ^a^	Stability in DMSO/RPMI-1640 (*v*/*v*) ^b^
**4a**	<15% degradation (3:2)	**4a** + other species ^c^ (1:3)
**4b**	ca. 50% degradation (1:1)	**4b** (1:3)
**4c**	<15% degradation (2:1)	**2c** + **4c** (1:4)
**4d**	<15% degradation (1:1)	**4d** (1:2)
**5a**		**5a** (1:1)
**6**		**6** (1:1)

^a^ After 24 h at 37 °C (^1^H NMR), [NaCl] ≈ 0.05 M. ^b^ Compounds detected (IR) in CH_2_Cl_2_ phase after 72 h at 37 °C. ^c^ Bands at 2097m, 1990m-s (**4a**), 1954m-sh, 1895w, 1814m (**4a**), 1670w-sh (**4a**), 1631s cm^−1^.

## Supplementary Materials

The following are available online: DFT structures, cyclic voltammograms, NMR spectra of products (signals around 0 ppm due to some silicon grease). CCDC reference number 1983327 (**3**) contains the supplementary crystallographic data for the X-ray studies reported in this paper. These data can be obtained free of charge at www.ccdc.cam.ac.uk/conts/retrieving.html (or from the Cambridge Crystallographic Data Centre, 12, Union Road, Cambridge CB2 1EZ, UK; fax: (internat.) +44-1223/336-033; e-mail: deposit@ccdc.cam.ac.uk).
