# Supplementary material for: Mono-, Di- and Tetra-iron Complexes with Selenium or Sulphur Functionalized Vinyliminium Ligands: Synthesis, Structural Characterization and Antiproliferative Activity
Source: Molecules. 2020 Apr 3;25(7):1656. doi: 10.3390/molecules25071656 (PMC7180837; doi:10.3390/molecules25071656)

checkCIF/PLATON report

Structure factors have been supplied for datablock(s) 3

THIS REPORT IS FOR GUIDANCE ONLY. IF USED AS PART OF A REVIEW PROCEDURE FOR PUBLICATION, IT SHOULD NOT REPLACE THE EXPERTISE OF AN EXPERIENCED CRYSTALLOGRAPHIC REFEREE.

No syntax errors found. CIF dictionary Interpreting this report

Datablock: 3

```
Bond precision:  C-C = 0.0675 A          Wavelength=0.71073

Cell:            a=13.454(3)     b=7.675(2)        c=12.285(2)
                 alpha=90        beta=99.07(3)     gamma=90
Temperature:     100 K

                Calculated                 Reported
Volume          1252.7(5)                  1252.5(5)
Space group     P c                        P c
Hall group      P -2yc                     P -2yc
Moiety formula  C12 H15 Fe N O Se          ?
Sum formula     C12 H15 Fe N O Se          C12 H15 Fe N O Se
Mr              324.06                     324.06
Dx,g cm-3       1.718                      1.719
Z               4                          4
Mu (mm-1)       4.087                      4.088
F000            648.0                      648.0
F000'           649.21
h,k,lmax        15,9,14                    15,9,14
Nref            4399[ 2204]                2119
Tmin,Tmax       0.441,0.542                0.355,0.745
Tmin'           0.408

Correction method= # Reported T Limits: Tmin=0.355 Tmax=0.745
AbsCorr = EMPIRICAL

Data completeness= 0.96/0.48      Theta(max)= 24.999

R(reflections)= 0.1174( 1870)     wR2(reflections)= 0.2914( 2119)

S = 1.116                 Npar= 290
```

The following ALERTS were generated. Each ALERT has the format
test-name_ALERT_alert-type_alert-level.
Click on the hyperlinks for more details of the test.

Alert level B

```
PLAT341_ALERT_3_B Low Bond Precision on  C-C Bonds ...............     0.0675 Ang.
```

Alert level C

```
PLAT082_ALERT_2_C High R1 Value ..................................       0.12 Report
PLAT084_ALERT_3_C High wR2 Value (i.e. > 0.25) ...................       0.29 Report
PLAT090_ALERT_3_C Poor Data / Parameter Ratio (Zmax > 18) ........       7.30 Note
PLAT147_ALERT_1_C s.u. on Symmetry Constrained Cell Angle(s) .....     Please Check
PLAT213_ALERT_2_C Atom C3              has ADP max/min Ratio .....        4.0 oblate
PLAT369_ALERT_2_C Long   C(sp2)-C(sp2) Bond  C28      - C29      .       1.56 Ang.
PLAT906_ALERT_3_C Large K Value in the Analysis of Variance ......      2.534 Check
PLAT911_ALERT_3_C Missing FCF Refl Between Thmin & STh/L=    0.595         87 Report
PLAT914_ALERT_3_C No Bijvoet Pairs in FCF for Non-centro Structure     Please Check
```

Alert level G

```
PLAT003_ALERT_2_G Number of Uiso or Uij Restrained non-H Atoms ...         32 Report
PLAT083_ALERT_2_G SHELXL Second Parameter in WGHT  Unusually Large     143.84 Why ?
PLAT178_ALERT_4_G The CIF-Embedded .res File Contains SIMU Records          1 Report
PLAT186_ALERT_4_G The CIF-Embedded .res File Contains ISOR Records          2 Report
PLAT380_ALERT_4_G Incorrectly? Oriented X(sp2)-Methyl Moiety .....         C5 Check
PLAT380_ALERT_4_G Incorrectly? Oriented X(sp2)-Methyl Moiety .....         C6 Check
PLAT380_ALERT_4_G Incorrectly? Oriented X(sp2)-Methyl Moiety .....         C7 Check
PLAT380_ALERT_4_G Incorrectly? Oriented X(sp2)-Methyl Moiety .....        C25 Check
PLAT380_ALERT_4_G Incorrectly? Oriented X(sp2)-Methyl Moiety .....        C26 Check
PLAT380_ALERT_4_G Incorrectly? Oriented X(sp2)-Methyl Moiety .....        C27 Check
PLAT860_ALERT_3_G Number of Least-Squares Restraints .............        350 Note
PLAT870_ALERT_4_G ALERTS Related to Twinning Effects Suppressed ..          ! Info
PLAT883_ALERT_1_G No Info/Value for _atom_sites_solution_primary .     Please Do !
PLAT909_ALERT_3_G Percentage of I>2sig(I) Data at Theta(Max) Still        84% Note
```

```
   0 ALERT level A = Most likely a serious problem - resolve or explain
   1 ALERT level B = A potentially serious problem, consider carefully
   9 ALERT level C = Check. Ensure it is not caused by an omission or oversight
  14 ALERT level G = General information/check it is not something unexpected

   2 ALERT type 1 CIF construction/syntax error, inconsistent or missing data
   5 ALERT type 2 Indicator that the structure model may be wrong or deficient
   8 ALERT type 3 Indicator that the structure quality may be low
   9 ALERT type 4 Improvement, methodology, query or suggestion
   0 ALERT type 5 Informative message, check
```

It is advisable to attempt to resolve as many as possible of the alerts in all categories. Often the minor alerts point to easily fixed oversights, errors and omissions in your CIF or refinement strategy, so attention to these fine details can be worthwhile. In order to resolve some of the more serious problems it may be necessary to carry out additional measurements or structure refinements. However, the purpose of your study may justify the reported deviations and the more serious of these should normally be commented upon in the discussion or experimental section of a paper or in the "special_details" fields of the CIF. checkCIF was carefully designed to identify outliers and unusual parameters, but every test has its limitations and alerts that are not important in a particular case may appear. Conversely, the absence of alerts does not guarantee there are no aspects of the results needing attention. It is up to the individual to critically assess their own results and, if necessary, seek expert advice.

Publication of your CIF in IUCr journals

A basic structural check has been run on your CIF. These basic checks will be run on all CIFs submitted for publication in IUCr journals (*Acta Crystallographica*, *Journal of Applied Crystallography*, *Journal of Synchrotron Radiation*); however, if you intend to submit to *Acta Crystallographica Section C* or *E* or *IUCrData*, you should make sure that full publication checks are run on the final version of your CIF prior to submission.

Publication of your CIF in other journals

Please refer to the *Notes for Authors* of the relevant journal for any special instructions relating to CIF submission.

PLATON version of 17/03/2019; check.def file version of 04/03/2019

Datablock 3 - ellipsoid plot

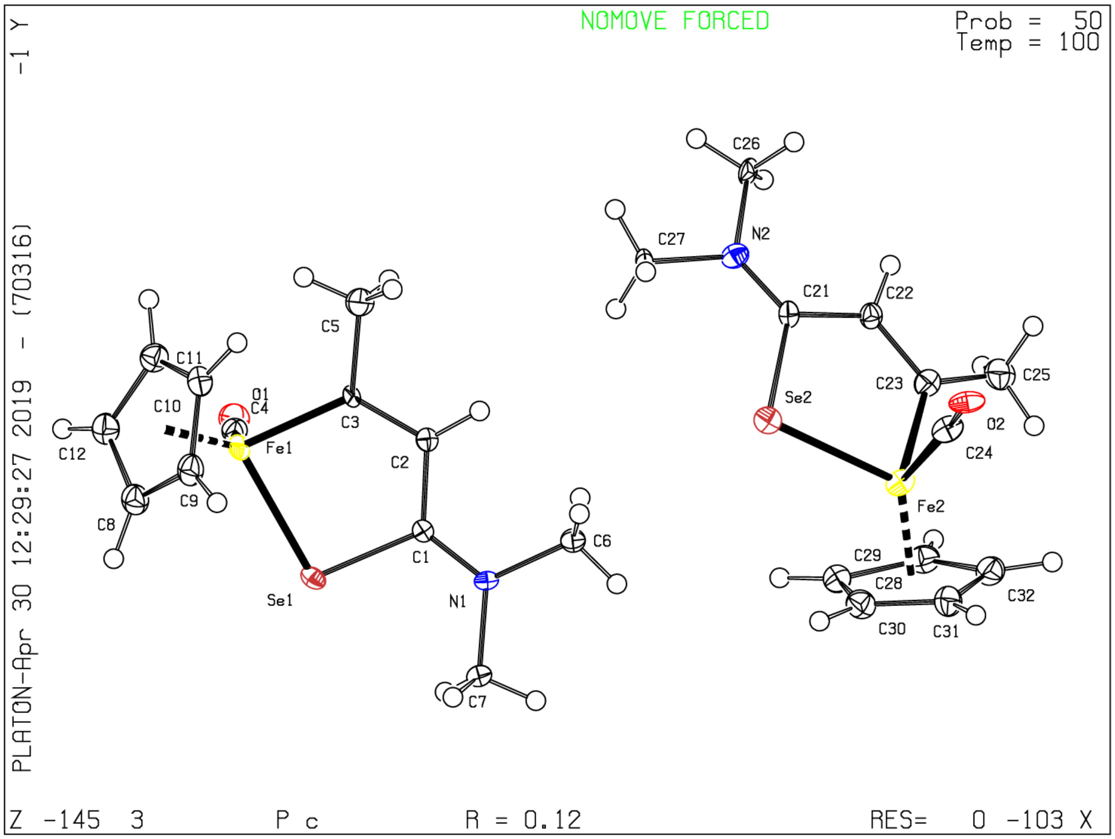

Supplement: Supplementary file 1 [file molecules-25-01656-s001.zip › Supporting Material/checkcif.pdf]
