# Supplementary material for: Mono-, Di- and Tetra-iron Complexes with Selenium or Sulphur Functionalized Vinyliminium Ligands: Synthesis, Structural Characterization and Antiproliferative Activity
Source: Molecules. 2020 Apr 3;25(7):1656. doi: 10.3390/molecules25071656 (PMC7180837; doi:10.3390/molecules25071656)

Mono-, Di- and Tetra-iron Complexes with Selenium or Sulphur Functionalized Vinyliminium Ligands: Synthesis, Structural Characterization and Antiproliferative Activity

Gabriele Agonigi, Lucinda K. Batchelor, Eleonora Ferretti, Silvia Schoch, Marco Bortoluzzi, Simona Braccini, Federica Chiellini, Lorenzo Biancalana, Stefano Zacchini, Guido Pampaloni, Biprajit Sarkar, Paul J. Dyson, Fabio Marchetti

Supporting Information

<u>Table of contents</u> *Pages*

Figures S1-S3: DFT structures S2

Figures S4-S14: Cyclic voltammograms S4

Figures S15-S34: NMR spectra S10

Figure S1. Electrostatic potential surfaces (isovalue = -20 kcal mol^{-1}) of the E-Z isomers of **2b**. EDF2/6-31G** calculations. Color map: Fe, green; S, yellow; O, red; N, blue; C, grey; H, white.

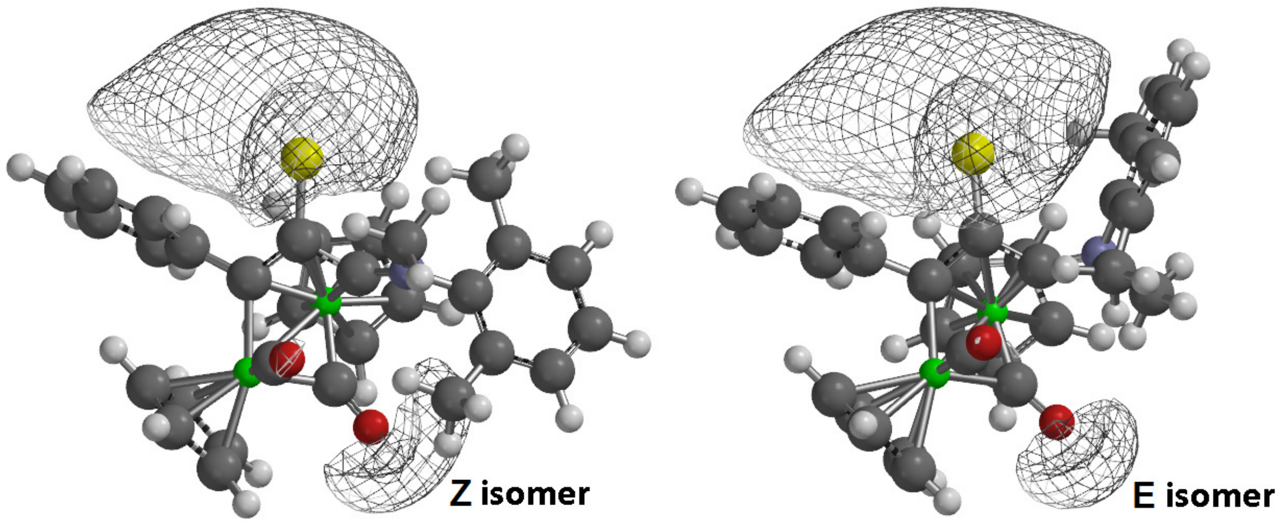

Figure S2. DFT-optimized structure of **4b** (Z configuration). C-PCM/ωB97X/def2-SVP calculation. Color map: Fe, green; S, yellow; O, red; N, blue; C, grey. Hydrogen atoms are omitted for clarity. Cartesian coordinates are collected in a separated .xyz file.

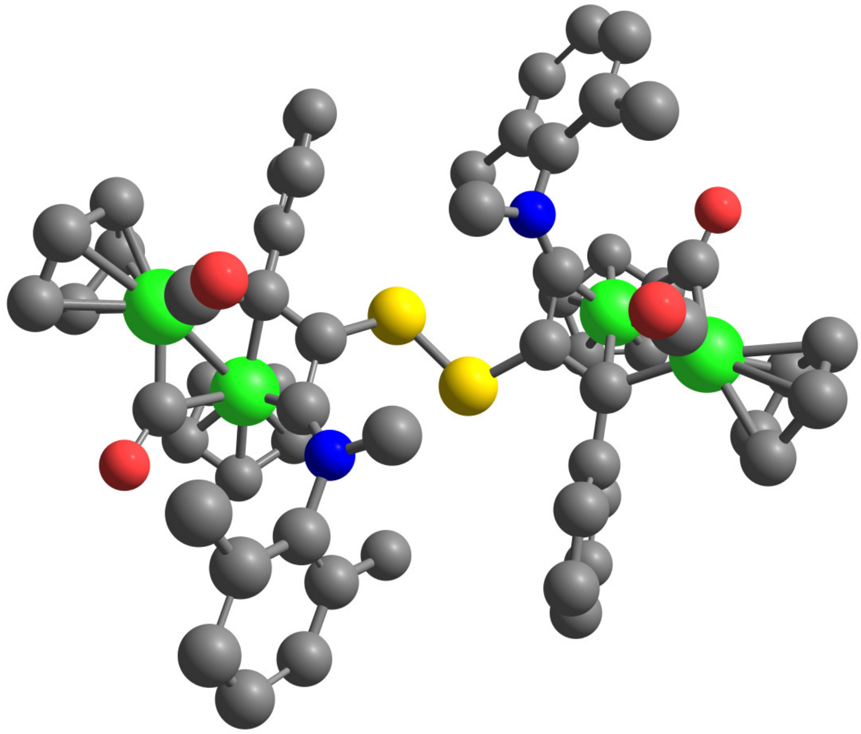

Figure S3. DFT-optimized structure of **4d** (Z configuration). C-PCM/ωB97X/def2-SVP calculation. Color map: Fe, green; Se, orange; O, red; N, blue; C, grey. Hydrogen atoms are omitted for clarity. Cartesian coordinates are collected in a separated .xyz file.

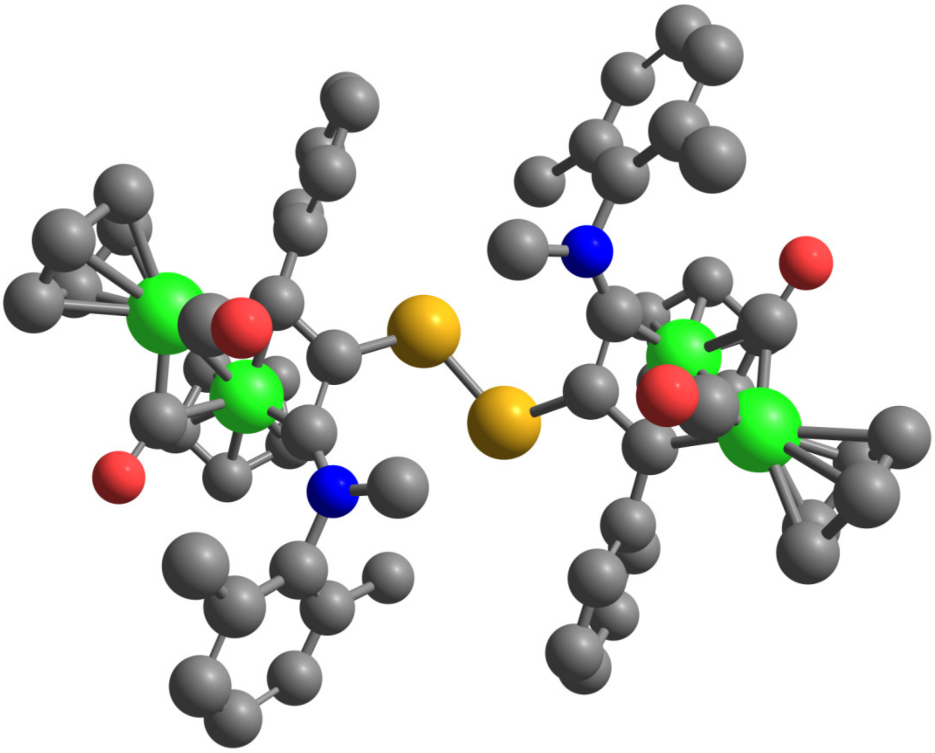

Figure S4. Cyclic voltammogram of **1a** recorded in MeCN at a scan rate of 100 mV/s with NBu_4PF_6 (0.1 M) as the supporting electrolyte; potentials reported versus the ferrocenium/ferrocene (Fc^+/Fc) redox couple.

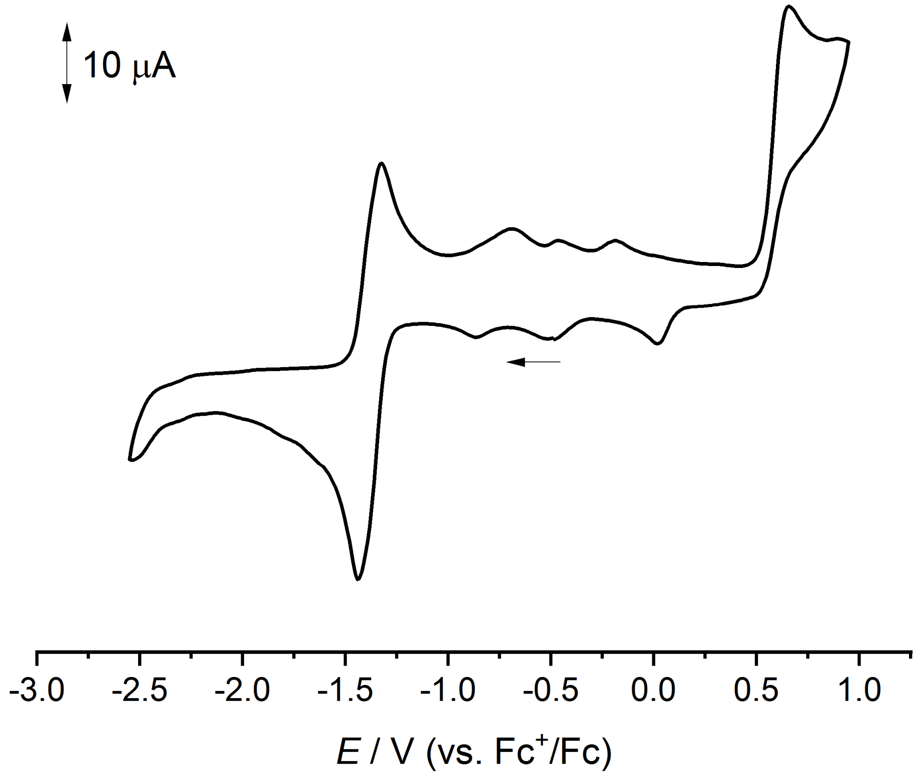

Figure S5. Cyclic voltammogram of the oxidation processes of **1a** recorded in MeCN at a scan rate of 25 mV/s (grey curve), 100 mV/s (red curve), and 500 mV/s (blue curve) with NBu_4PF_6 (0.1 M) as the supporting electrolyte; potentials reported versus the ferrocenium/ferrocene (Fc^+/Fc) redox couple.

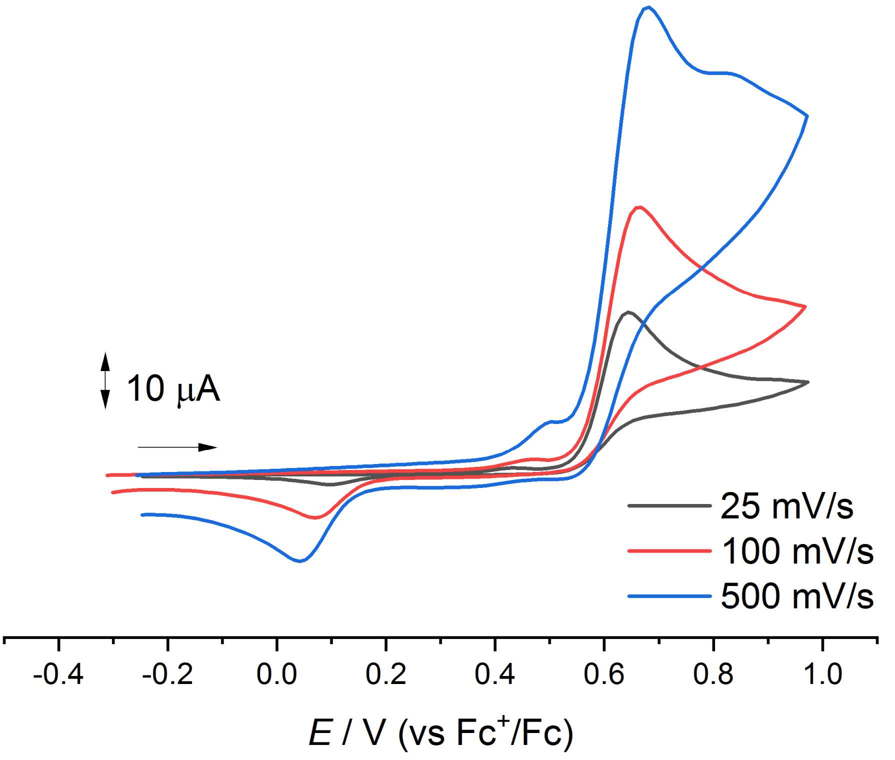

Figure S6. Cyclic voltammogram of the reduction processes of **1a** recorded in MeCN at a scan rate of 25 mV/s (grey curve), 100 mV/s (red curve), and 500 mV/s (blue curve) with NBu_4PF_6 (0.1 M) as the supporting electrolyte; potentials reported versus the ferrocenium/ferrocene (Fc^+/Fc) redox couple.

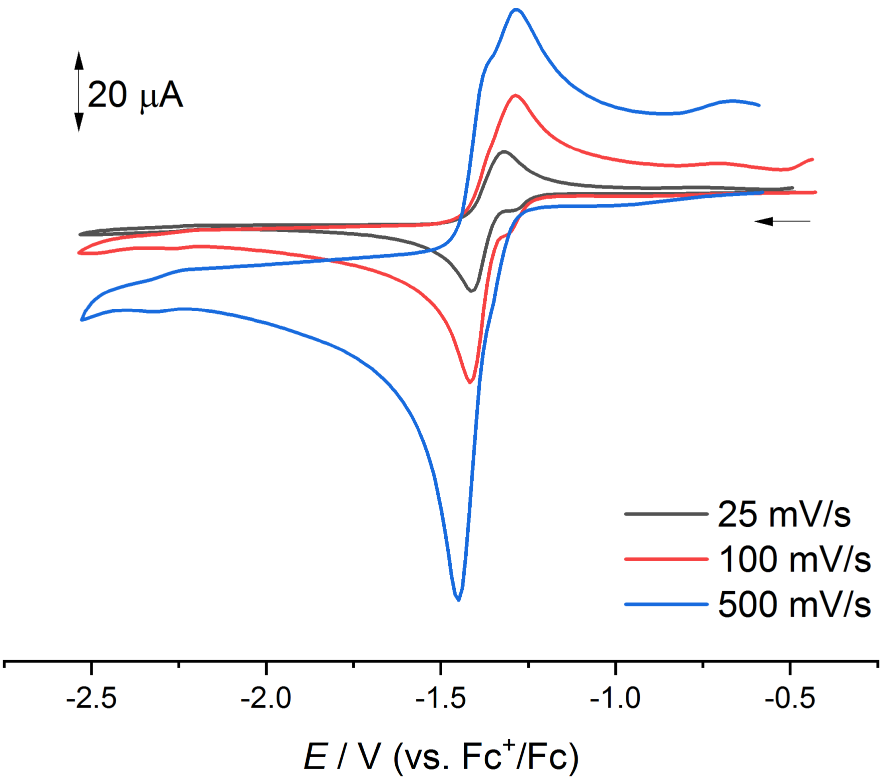

Figure S7. Cyclic voltammogram of **1c** recorded in MeCN at a scan rate of 100 mV/s with NBu_4PF_6 (0.1 M) as the supporting electrolyte; potentials reported versus the ferrocenium/ferrocene (Fc^+/Fc) redox couple.

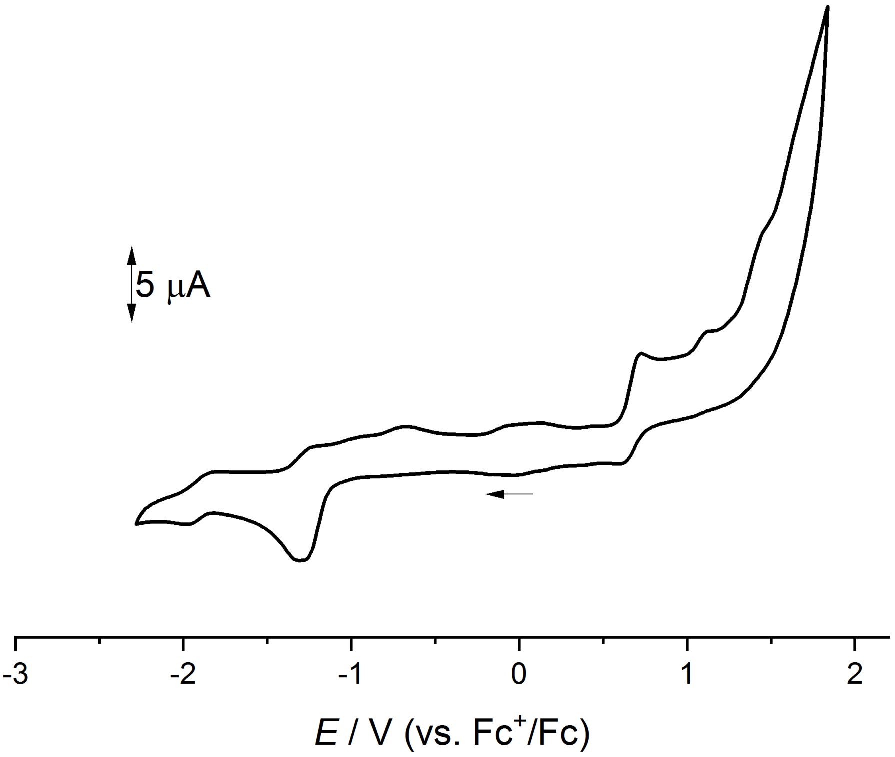

Figure S8. Cyclic voltammogram of **1c** recorded in MeCN at a scan rate of 25 mV/s (grey curve), 100 mV/s (red curve), and 500 mV/s (blue curve) with NBu_4PF_6 (0.1 M) as the supporting electrolyte; potentials reported versus the ferrocenium/ferrocene (Fc^+/Fc) redox couple.

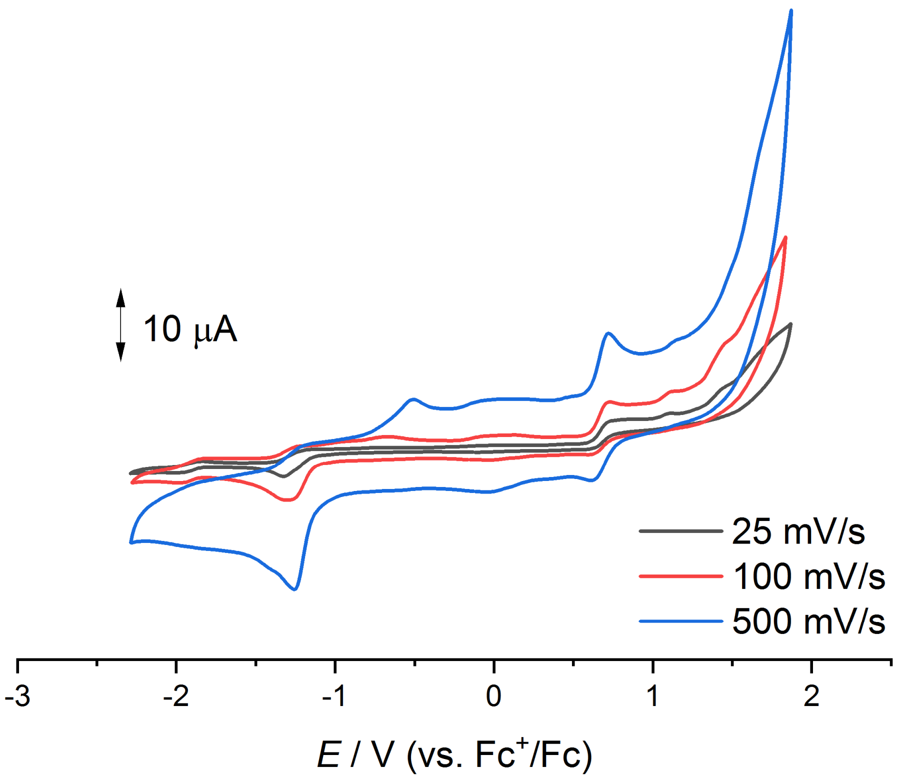

Figure S9. Cyclic voltammogram of **2c** recorded in MeCN at a scan rate of 100 mV/s with NBu_4PF_6 (0.1 M) as the supporting electrolyte; potentials reported versus the ferrocenium/ferrocene (Fc^+/Fc) redox couple.

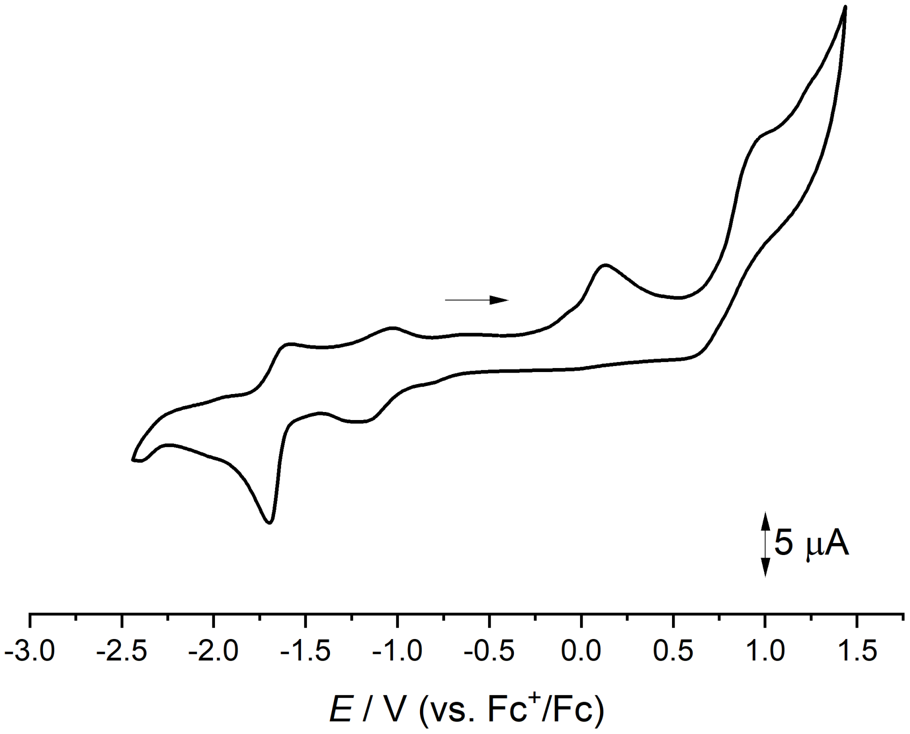

Figure S10. Cyclic voltammogram of **2c** recorded in MeCN at a scan rate of 25 mV/s (black curve), 100 mV/s (red curve), and 500 mV/s (blue curve) with NBu_4PF_6 (0.1 M) as the supporting electrolyte; potentials reported versus the ferrocenium/ferrocene (Fc^+/Fc) redox couple.

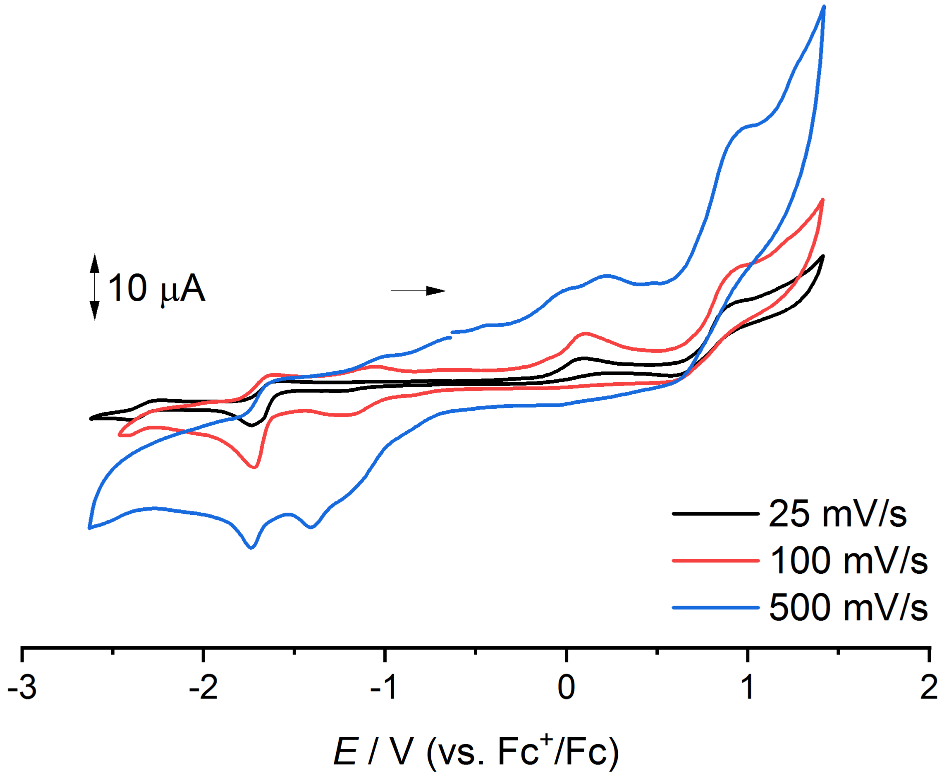

Figure S11. Cyclic voltammogram of the reduction of **4c** recorded in MeCN at a scan rate of 100 mV/s with NBu_4PF_6 (0.1 M) as the supporting electrolyte; potentials reported versus the ferrocenium/ferrocene (Fc^+/Fc) redox couple.

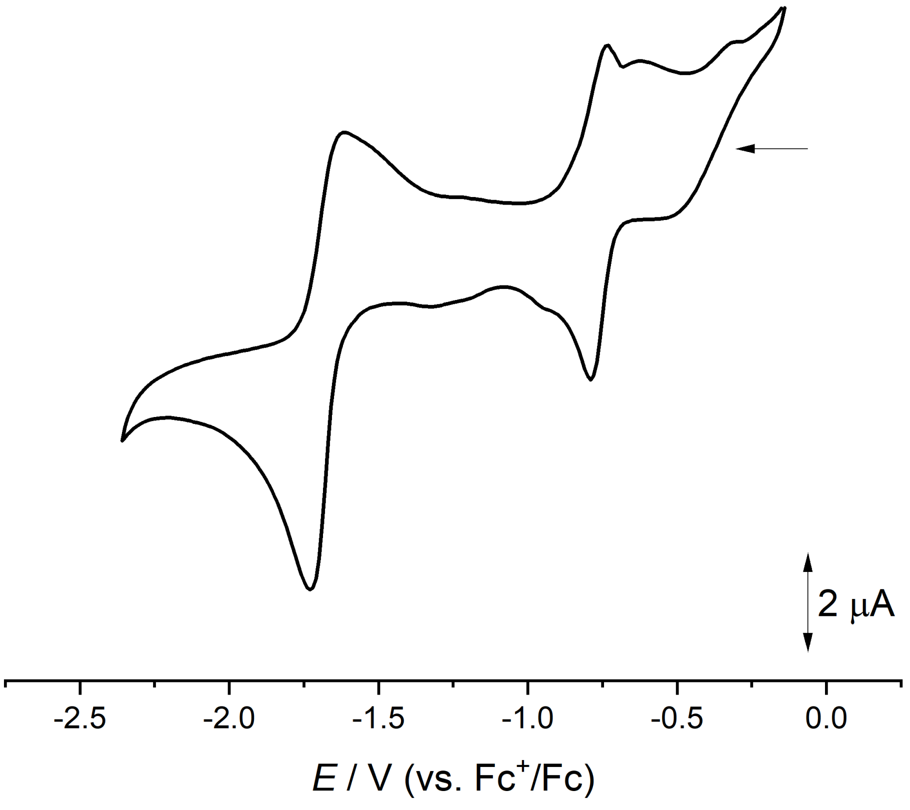

Figure S12. Cyclic voltammogram of **5a** recorded in MeCN at a scan rate of 100 mV/s with NBu_4PF_6 (0.1 M) as the supporting electrolyte (initial scan towards reduction and then oxidation); potentials reported versus the ferrocenium/ferrocene (Fc^+/Fc) redox couple.

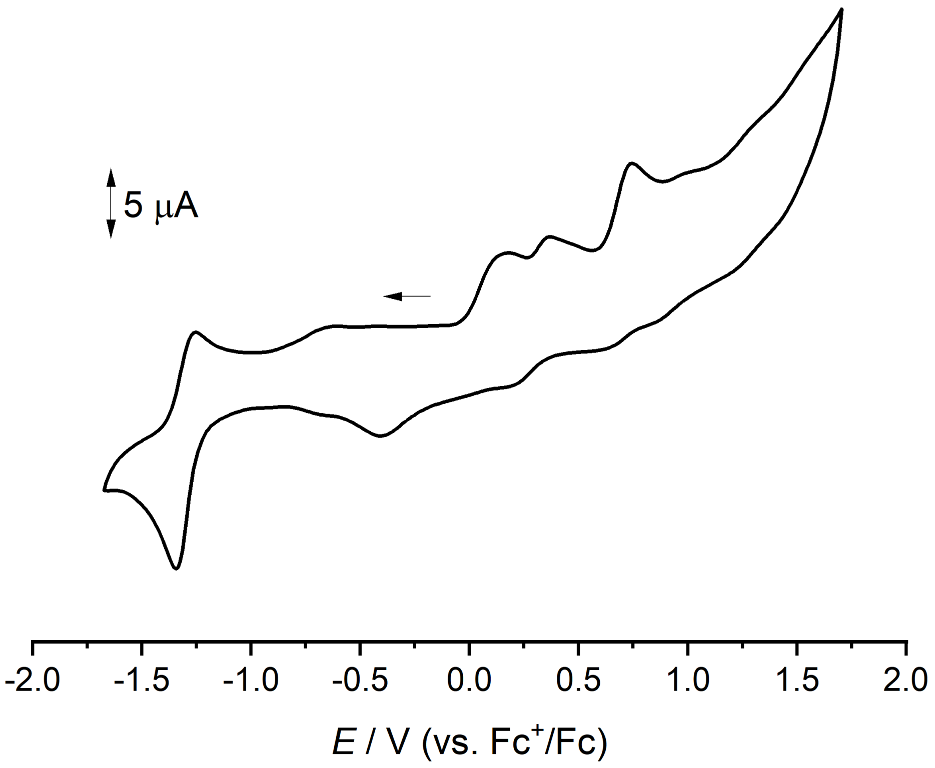

Figure S13. Cyclic voltammogram of **5a** recorded in MeCN at a scan rate of 100 mV/s with NBu_4PF_6 (0.1 M) as the supporting electrolyte (initial scan towards oxidation and then reduction); potentials reported versus the ferrocenium/ferrocene (Fc^+/Fc) redox couple.

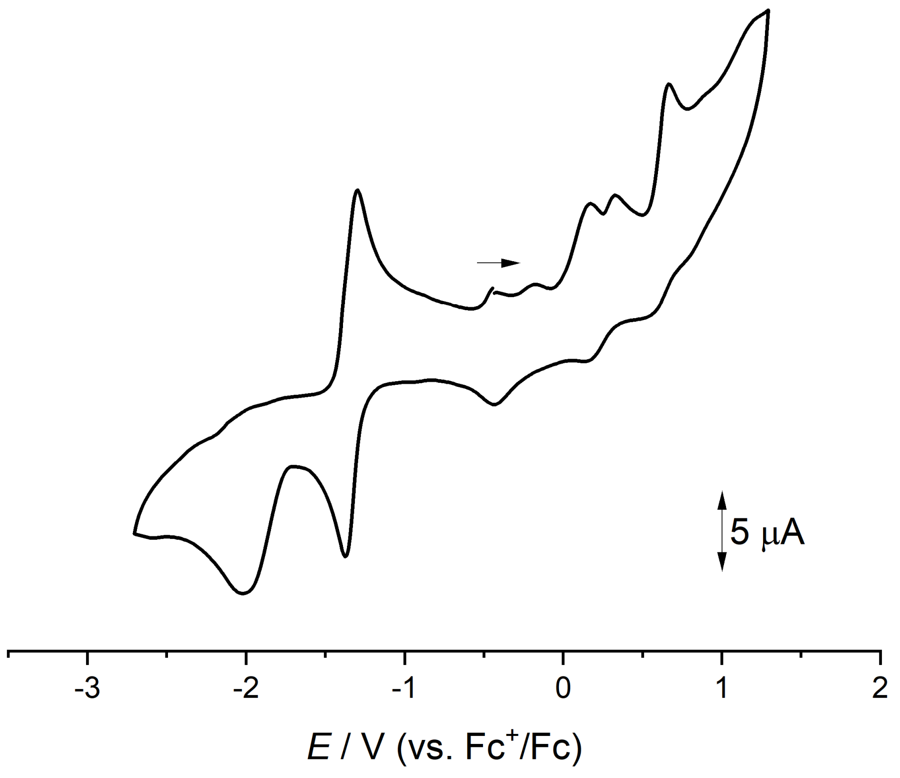

Figure S14. Cyclic voltammogram of **5a** (only reduction) recorded in MeCN at a scan rate of 25 mV/s (grey curve) and 100 mV/s (red curve) with NBu_4PF_6 (0.1 M) as the supporting electrolyte; potentials reported versus the ferrocenium/ferrocene (Fc^+/Fc) redox couple.

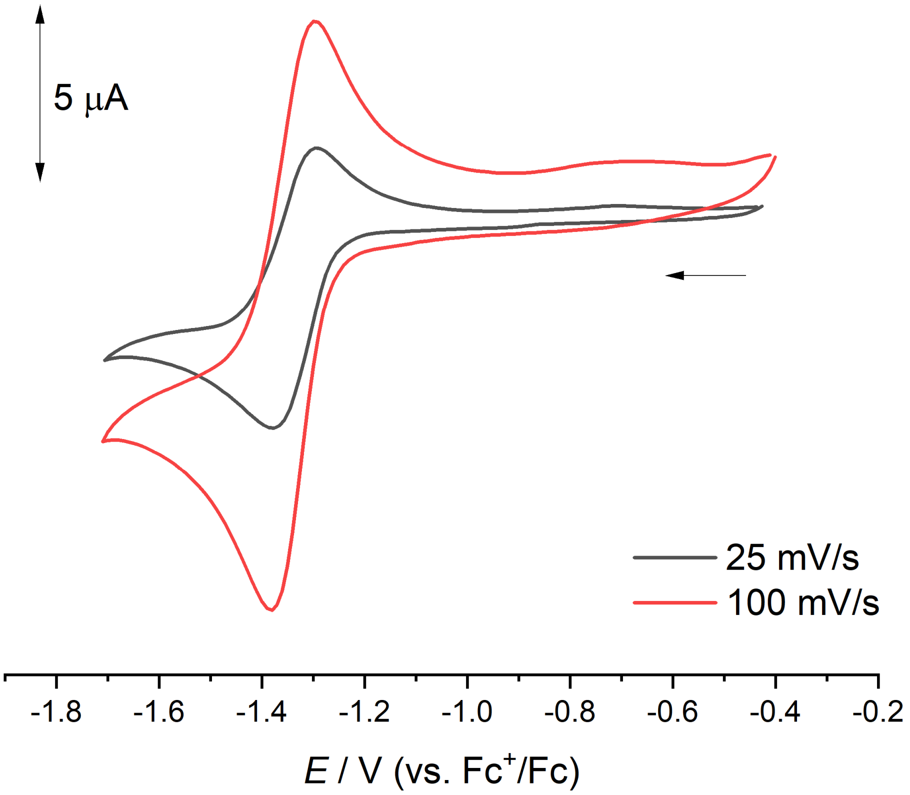

Figure S15. ^{1}H NMR spectrum (401 MHz, CDCl$_3$) of [Fe$_2$Cp$_2$(CO)(μ-CO){μ-η^{1}:η^{3}-C^{3}(Ph)C^{2}(S)C^{1}N(Me)(Xyl)}], **2b**.

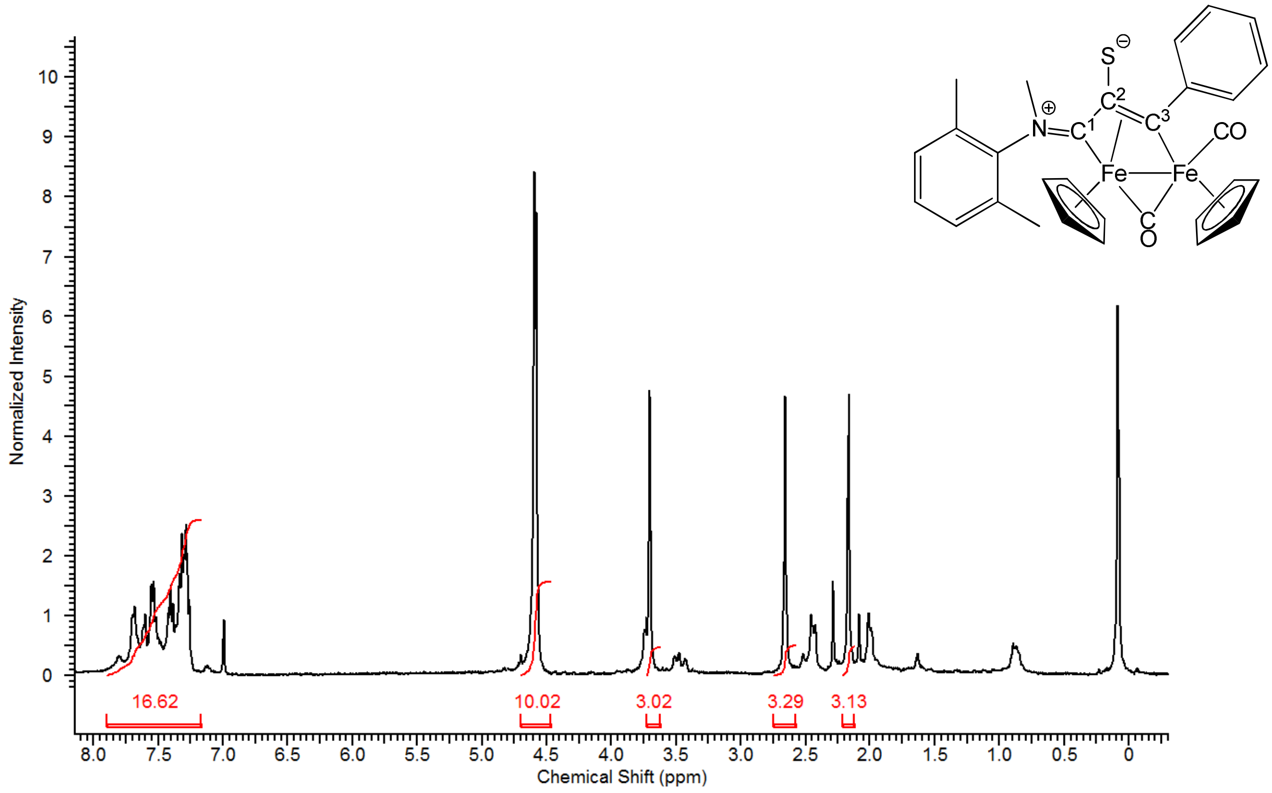

Figure S16. ^{13}C{^{1}H} NMR spectrum (101 MHz, CDCl$_3$) of **2b**.

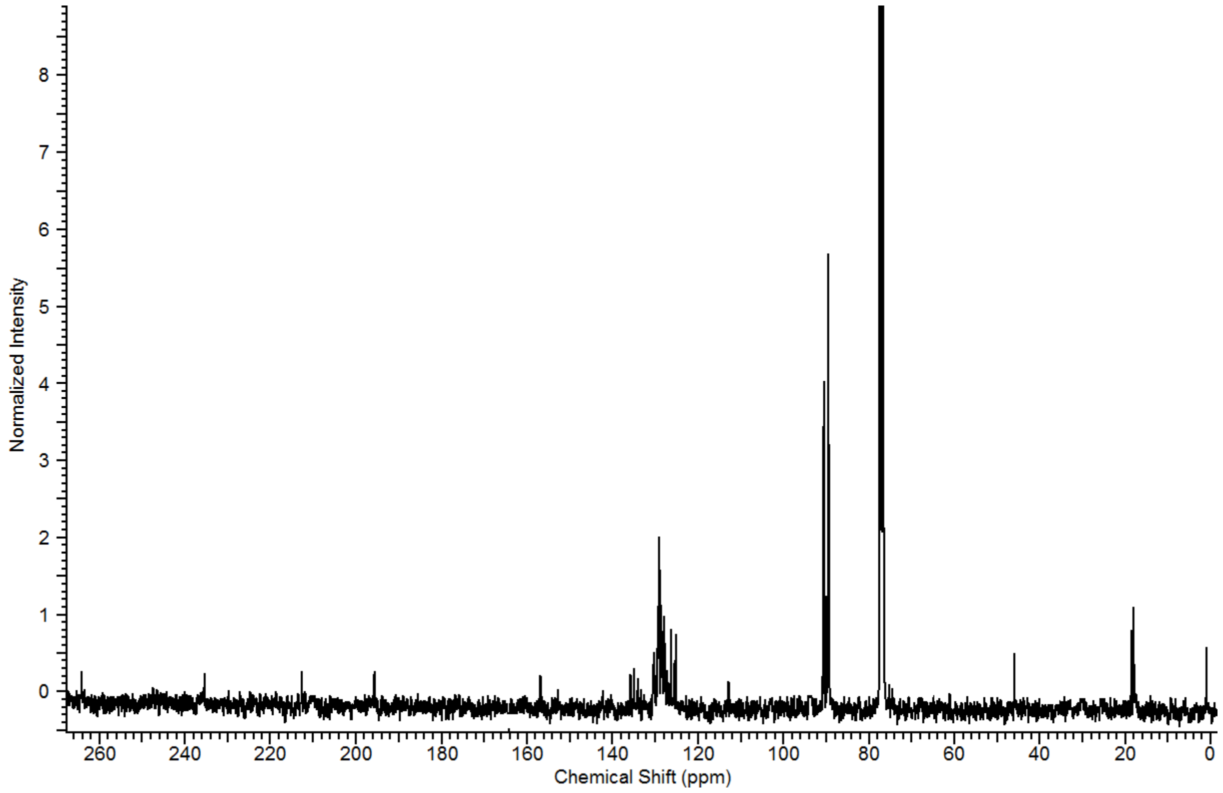

Figure S17. ^{1}H NMR spectrum (401 MHz, $CDCl_3$) of [$Fe_2Cp_2(CO)(\mu\text{-}CO)\{\mu\text{-}\eta^1{:}\eta^3\text{-}C^3(Ph)C^2(Se)C^1N(Me)(Xyl)\}$], **2d**.

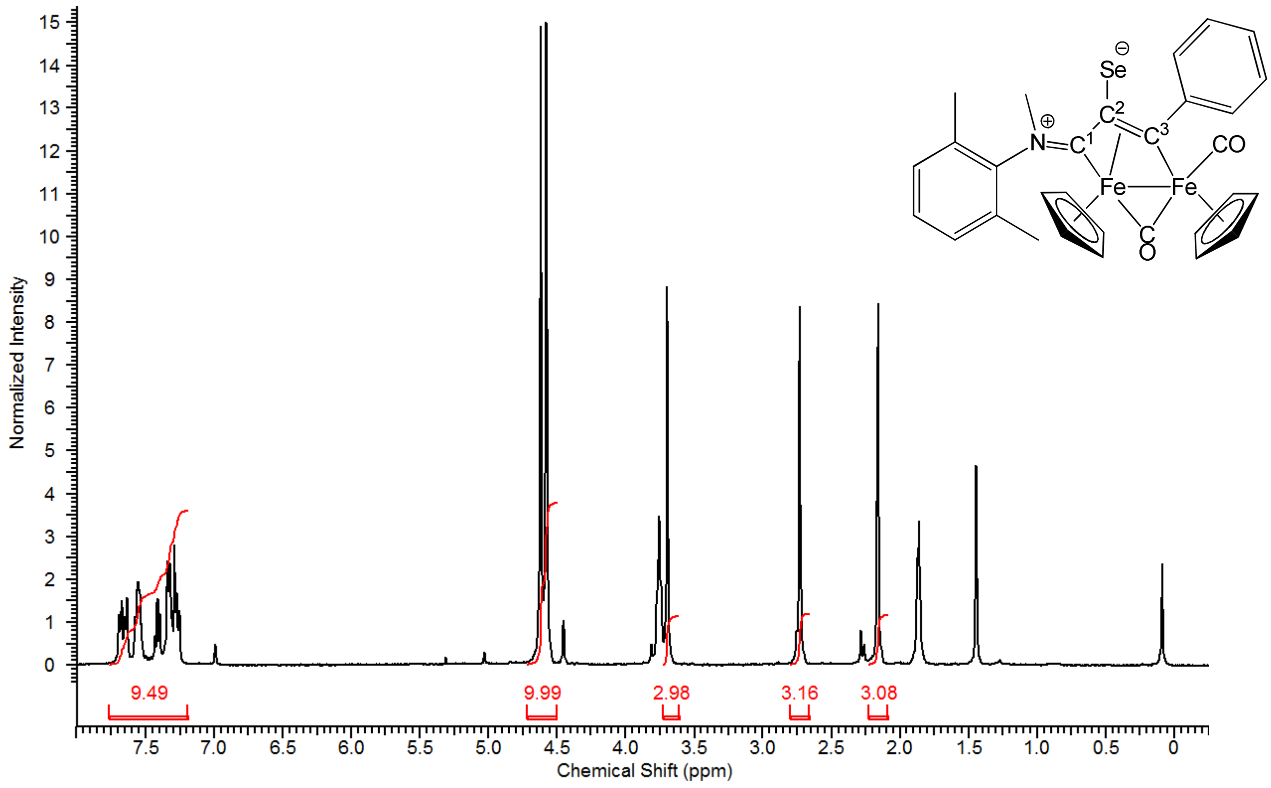

Figure S18. ^{13}C{^{1}H} NMR spectrum (101 MHz, $CDCl_3$) of **2d**.

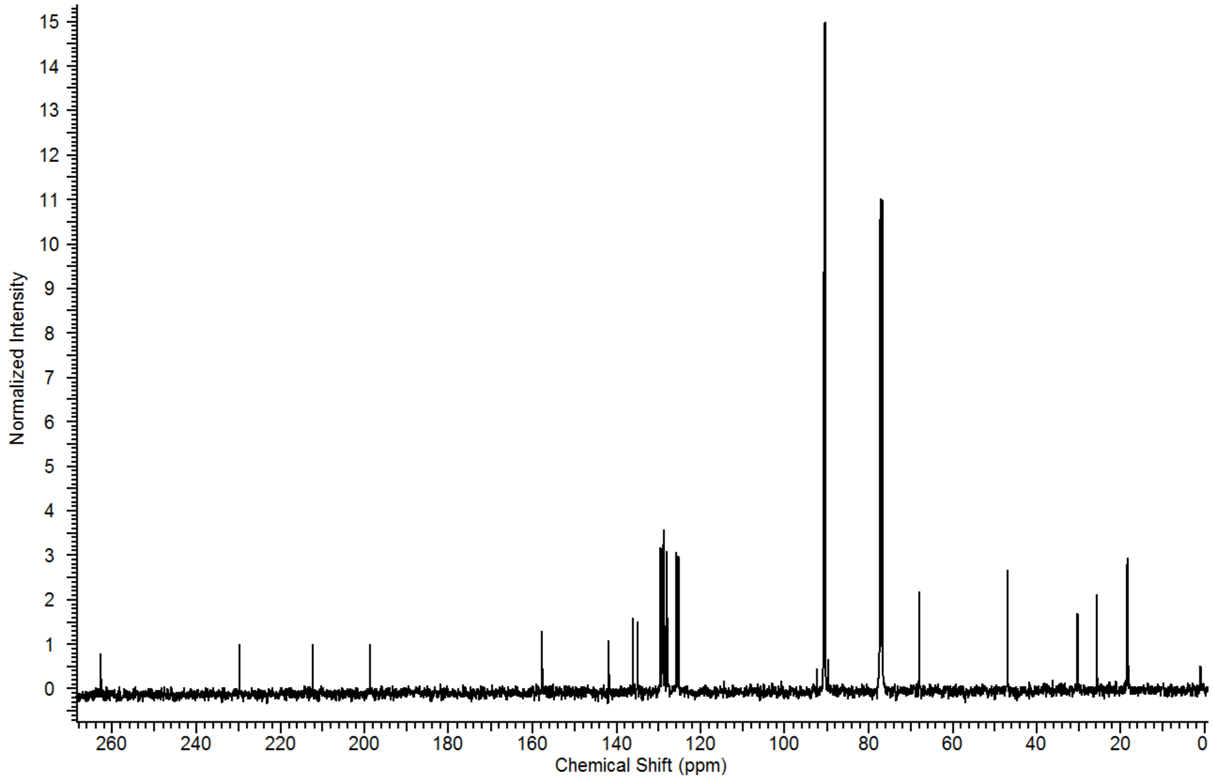

Figure S19. ^{77}Se NMR spectrum (76 MHz, $CDCl_3$) of **2d**.

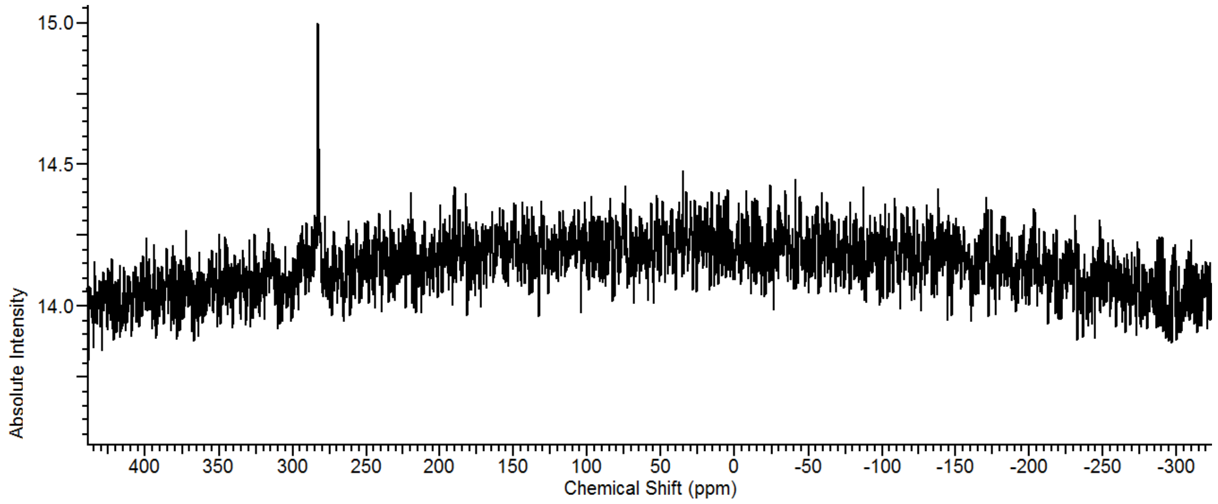

Figure S20. ^{1}H NMR spectrum (401 MHz, acetone-d_6) of [FeCp(CO){$C^1(NMe_2)C^2HC^3(Me)Se$}], **3**.

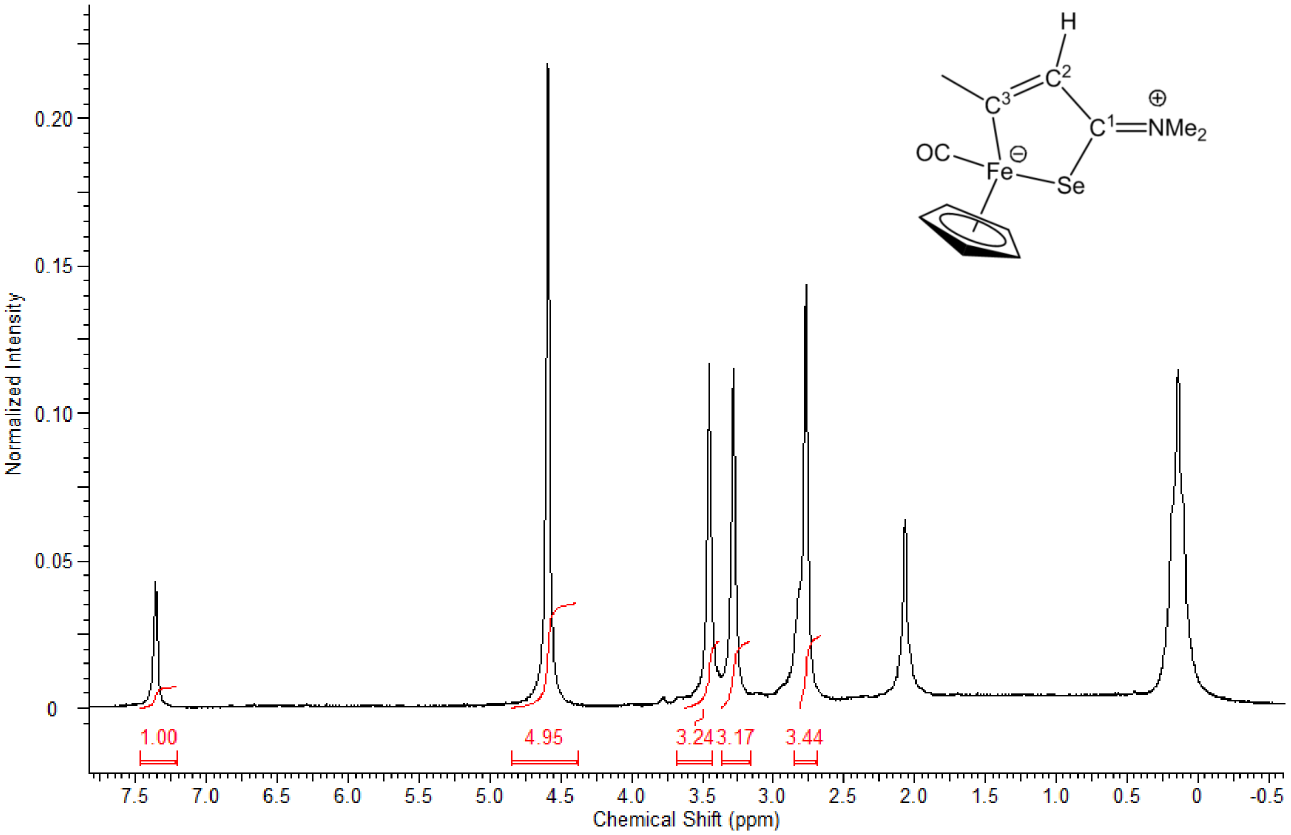

Figure S21. $^{13}C\{^{1}H\}$ NMR spectrum (101 MHz, acetone-d_6) of **3**.

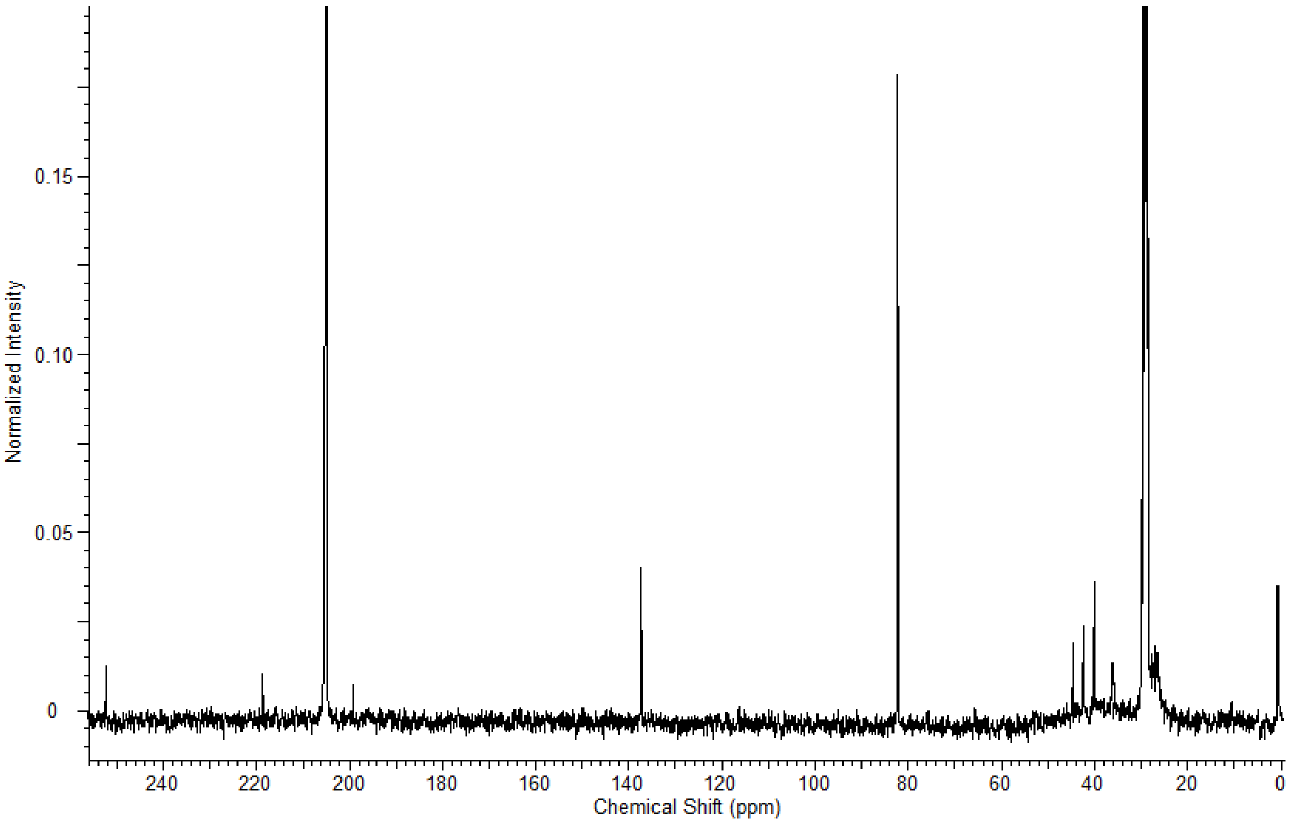

Figure S22. ^{77}Se NMR spectrum (76 MHz, acetone-d_6) of **3**.

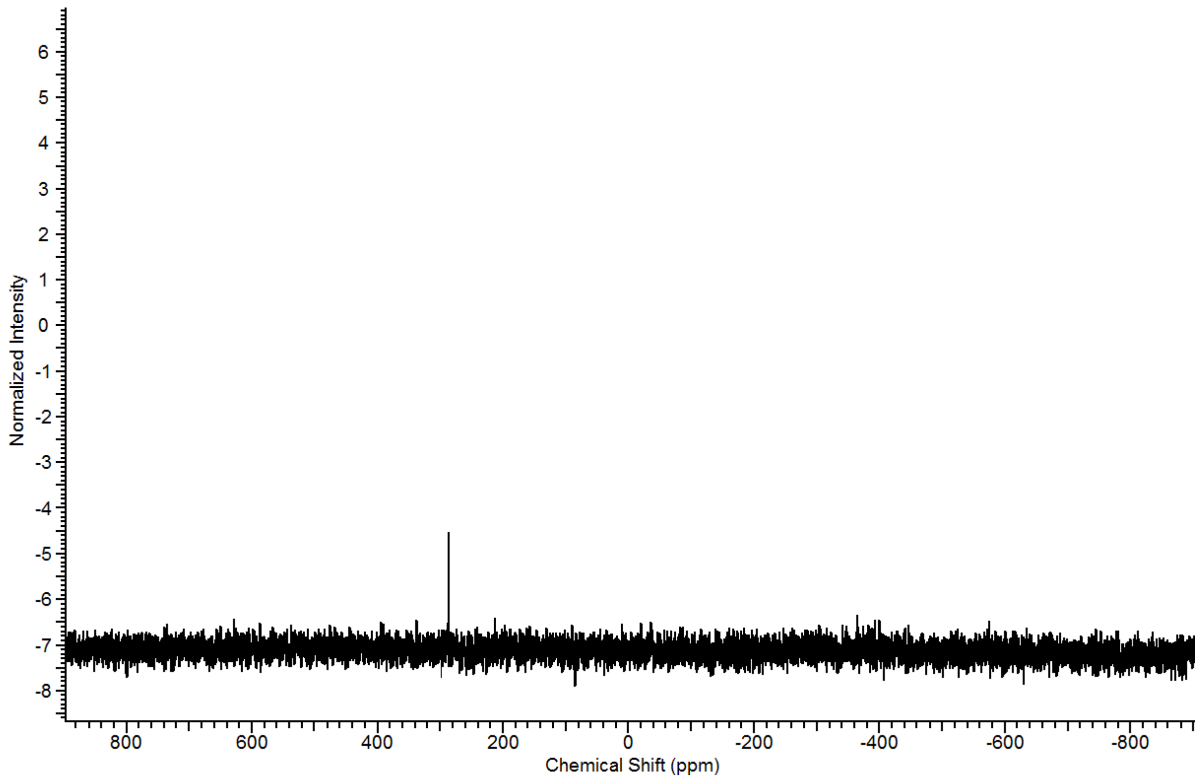

Normalized Intensity
6
5
4
3
2
1
0
-1
-2
-3
-4
-5
-6
-7
-8
800
600
400
200
0
-200
-400
-600
-800
Chemical Shift (ppm)

Figure S23. ^{1}H NMR spectrum (401 MHz, acetone-d$_6$) of $[Fe_2Cp_2(CO)(\mu\text{-}CO)\{\mu\text{-}\eta^1\text{:}\eta^3\text{-}C^3(Me)C^2(Se)C^1NMe_2\}]_2[I]_2$, **4a**.

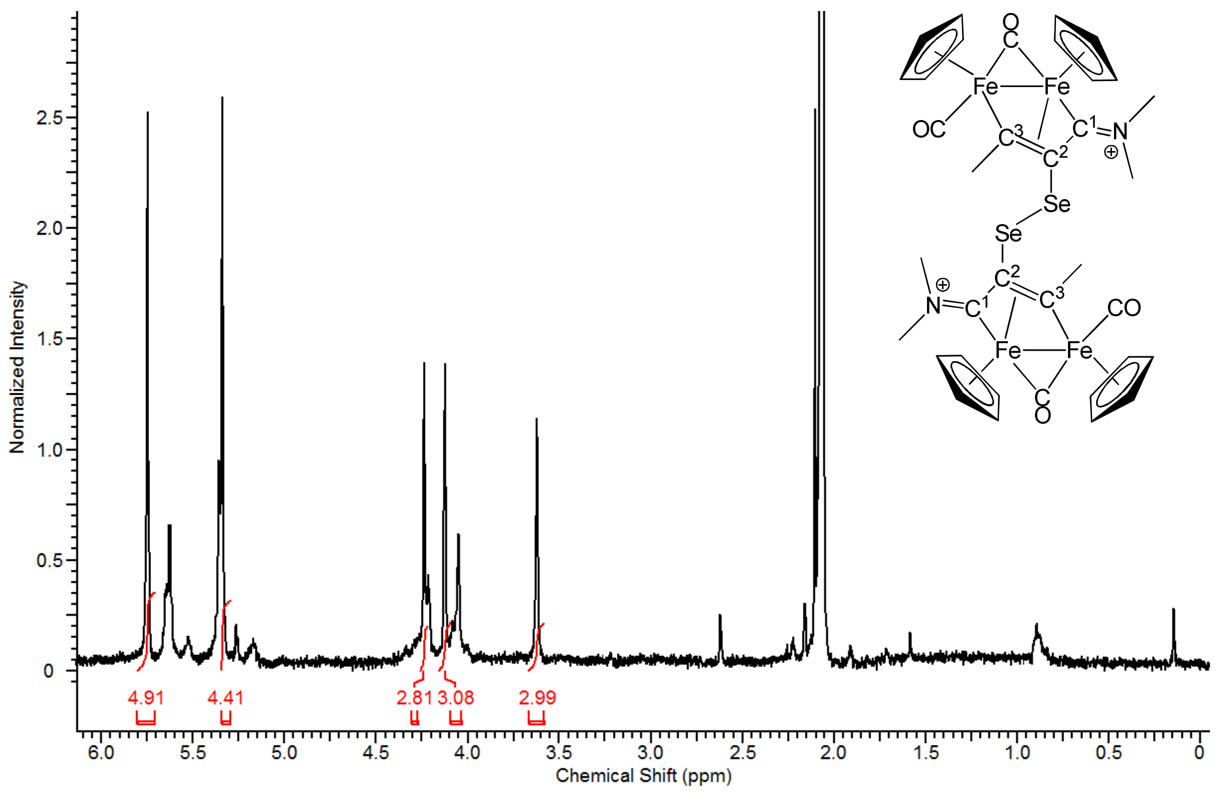

Figure S24. $^{13}C\{^1H\}$ NMR spectrum (101 MHz, acetone-d$_6$) of **4a**.

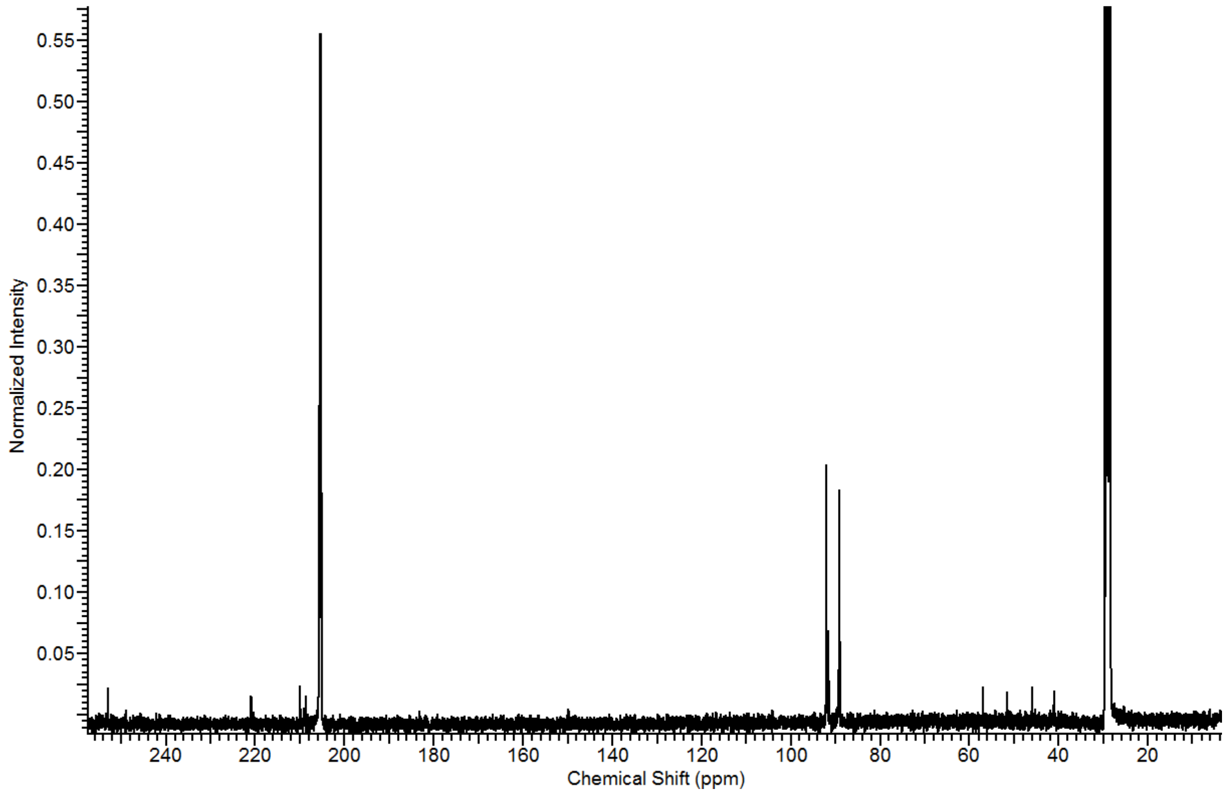

Figure S25. ^{77}Se NMR spectrum (76 MHz, dmso-d$_6$) of **4a**.

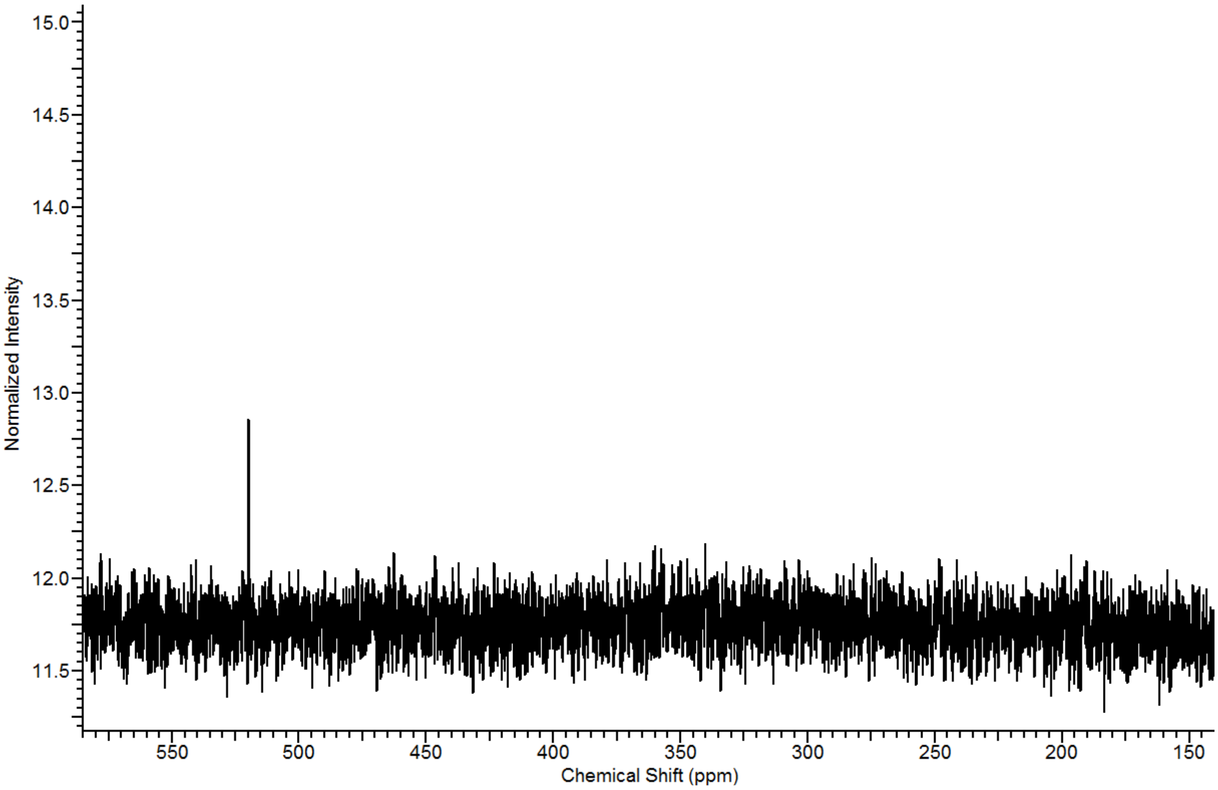

Figure S26. ^{1}H NMR spectrum (401 MHz, CD_2Cl_2) of $[Fe_2Cp_2(CO)(\mu\text{-}CO)\{\mu\text{-}\eta^1:\eta^3\text{-}C^3(Ph)C^2(S)C^1N(Me)(Xyl)\}]_2[I]_2$, **4b**.

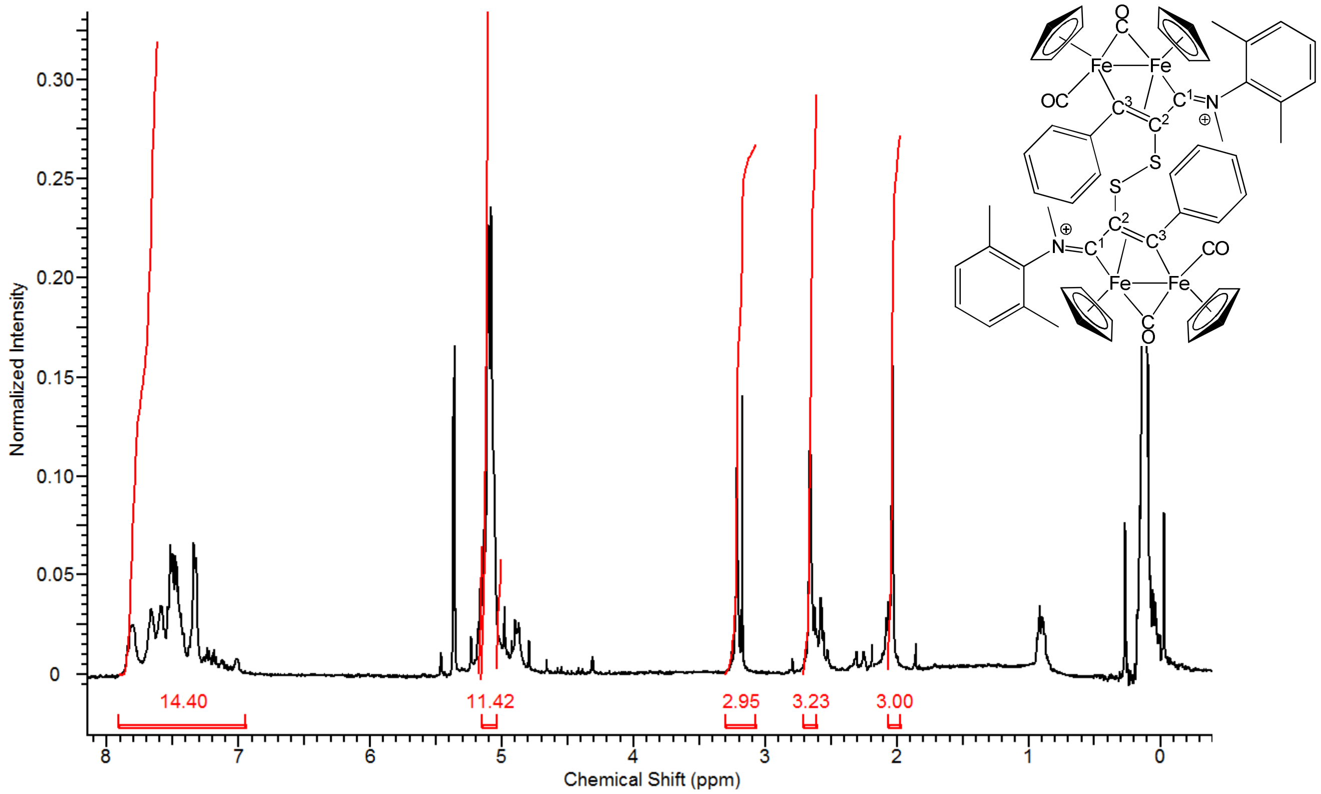

Figure S27. $^{13}C\{^{1}H\}$ NMR spectrum (101 MHz, CD_2Cl_2) of **4b**.

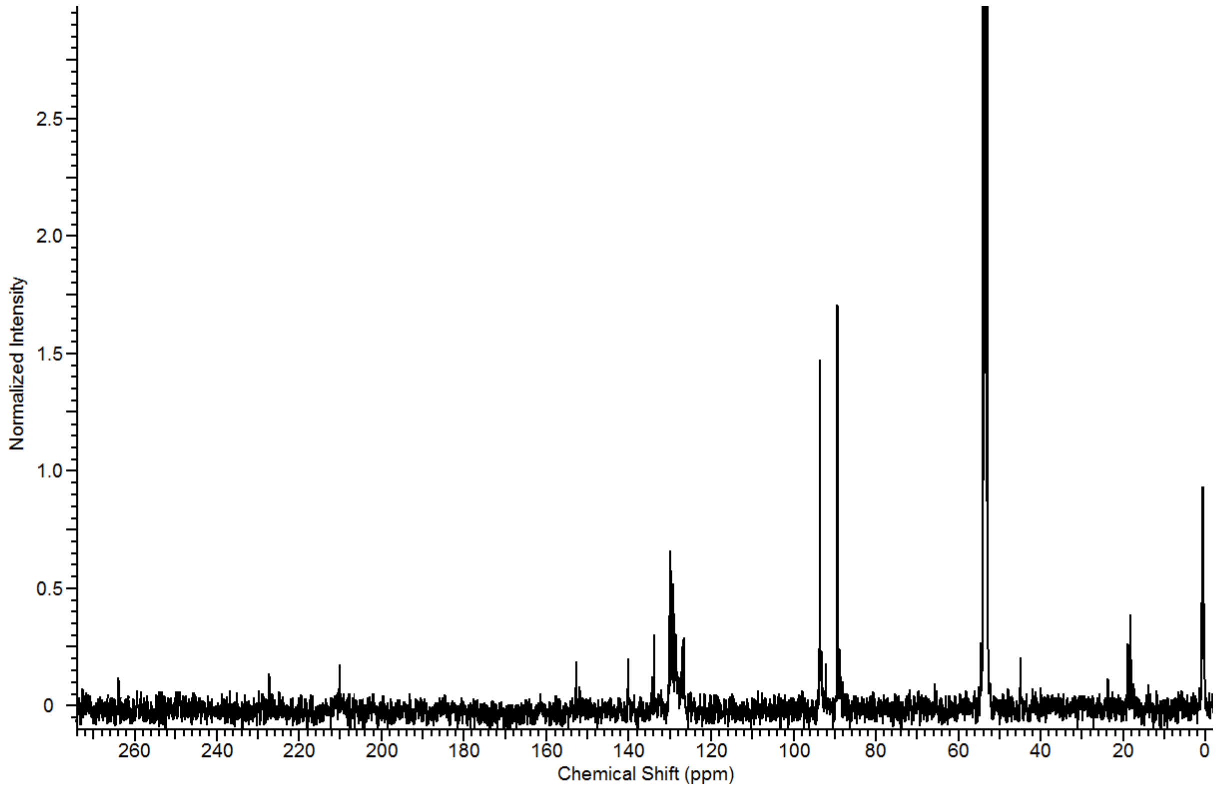

Figure S28. ¹H NMR spectrum (401 MHz, CD_3CN) of $[Fe_2Cp_2(CO)(\mu\text{-}CO)\{\mu\text{-}\eta^1:\eta^3\text{-}C^3(Ph)C^2(Se)C^1N(Me)(Xyl)\}]_2[I]_2$, **4d**.

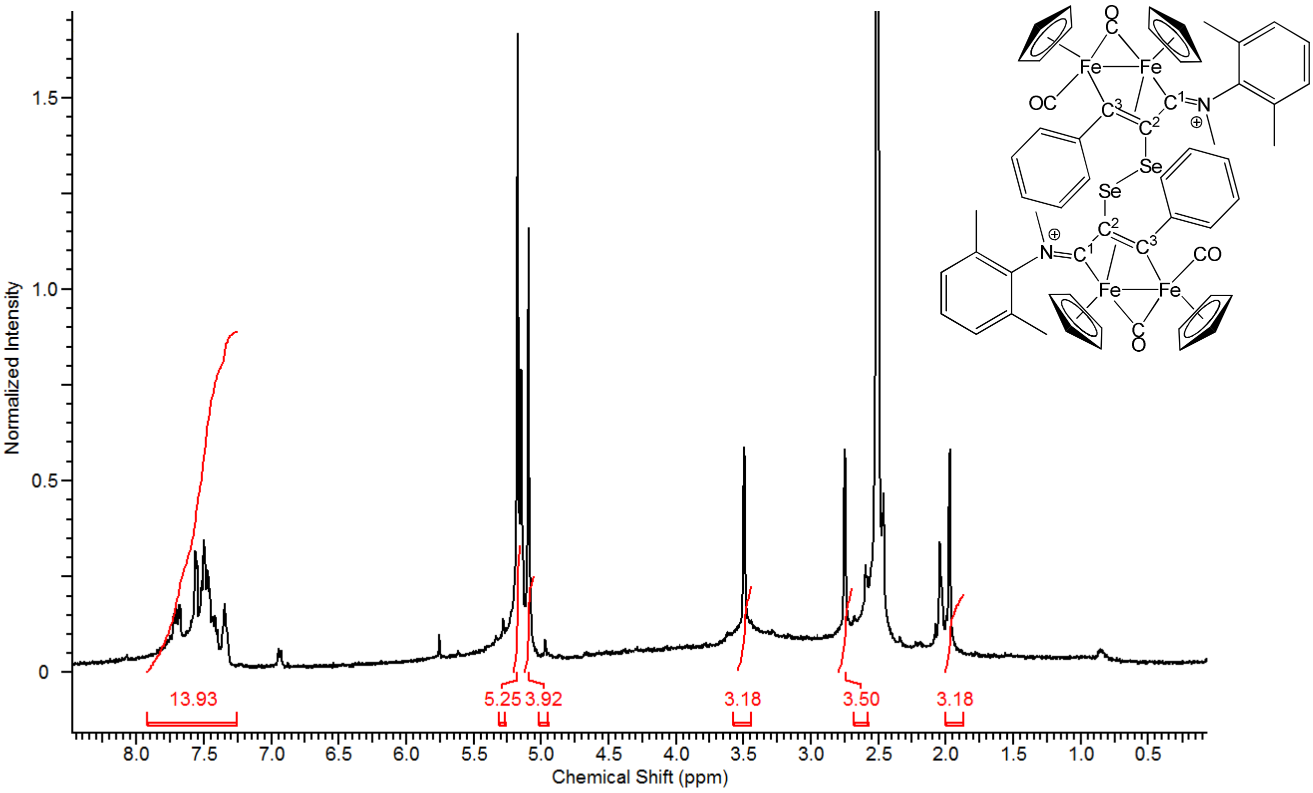

Figure S29. ^{77}Se NMR spectrum (76 MHz, dmso-d_6) of **4d**.

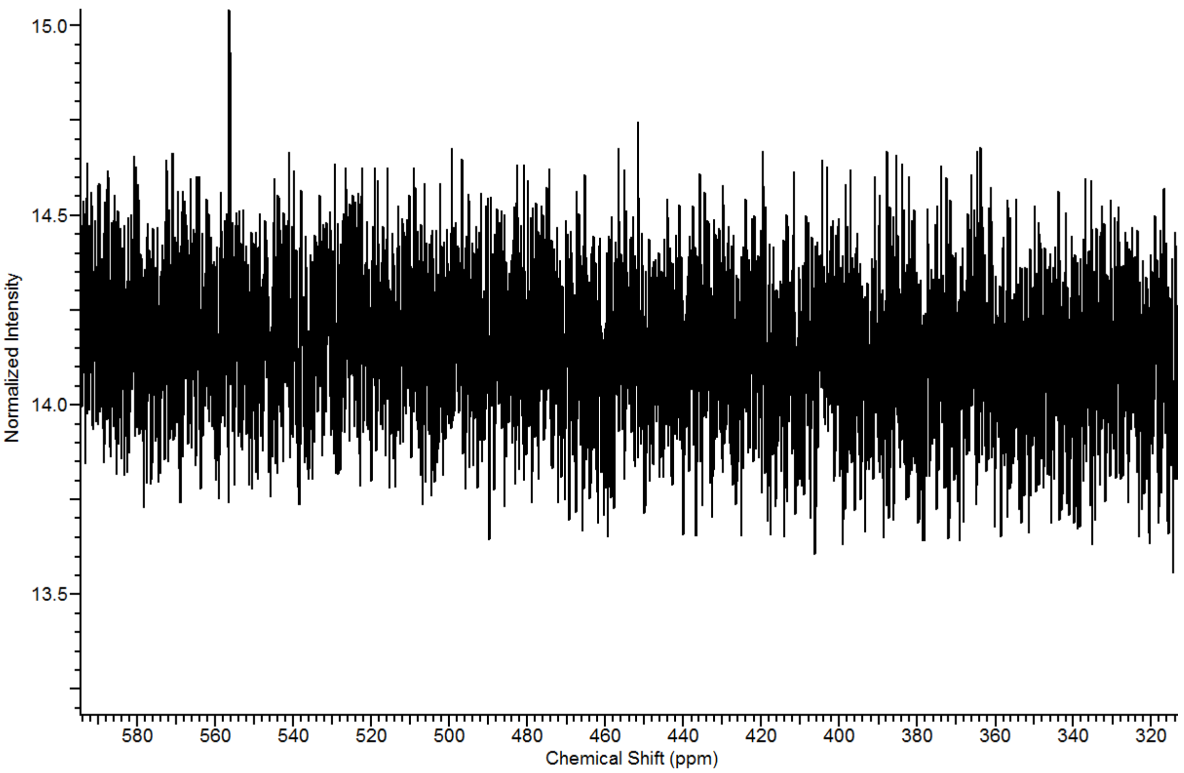

Figure S30. ^{1}H NMR spectrum (401 MHz, acetone-d_6) of $[Fe_2Cp_2(CO)(\mu\text{-}CO)\{\mu\text{-}\eta^1:\eta^3\text{-}C^3(Me)C^2(SeMe)C^1NMe_2\}]I$, **5a**.

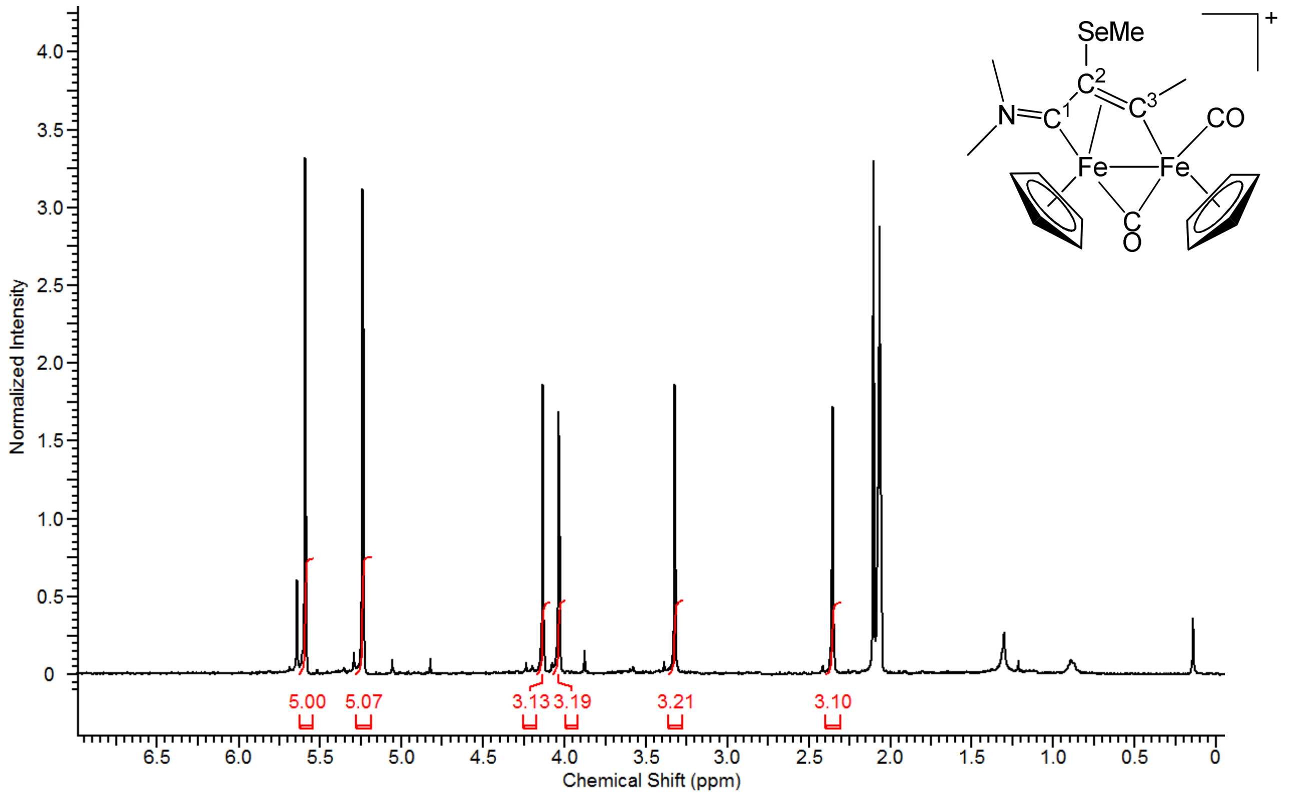

Figure S31. $^{13}C\{^{1}H\}$ NMR spectrum (101 MHz, acetone-d_6) of **5a**.

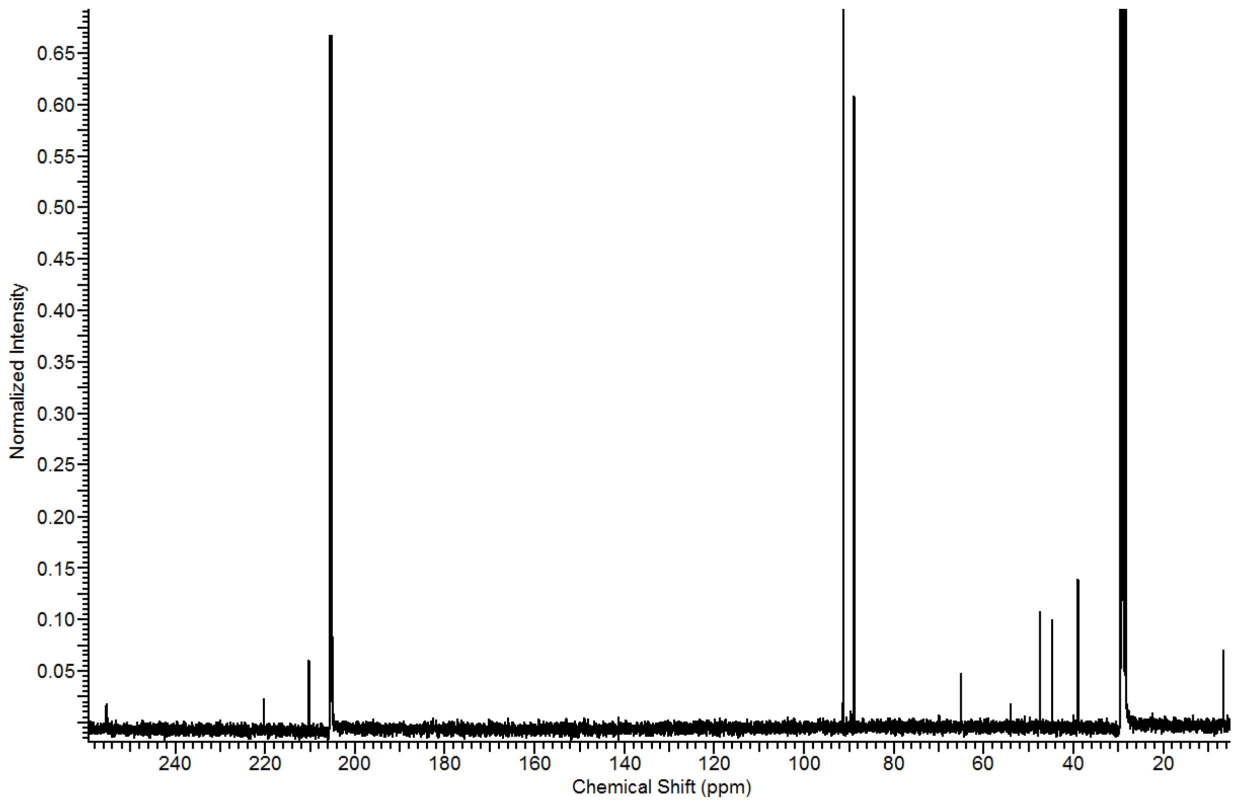

Figure S32. ^{77}Se NMR spectrum (76 MHz, dmso-d_6) of **5a**.

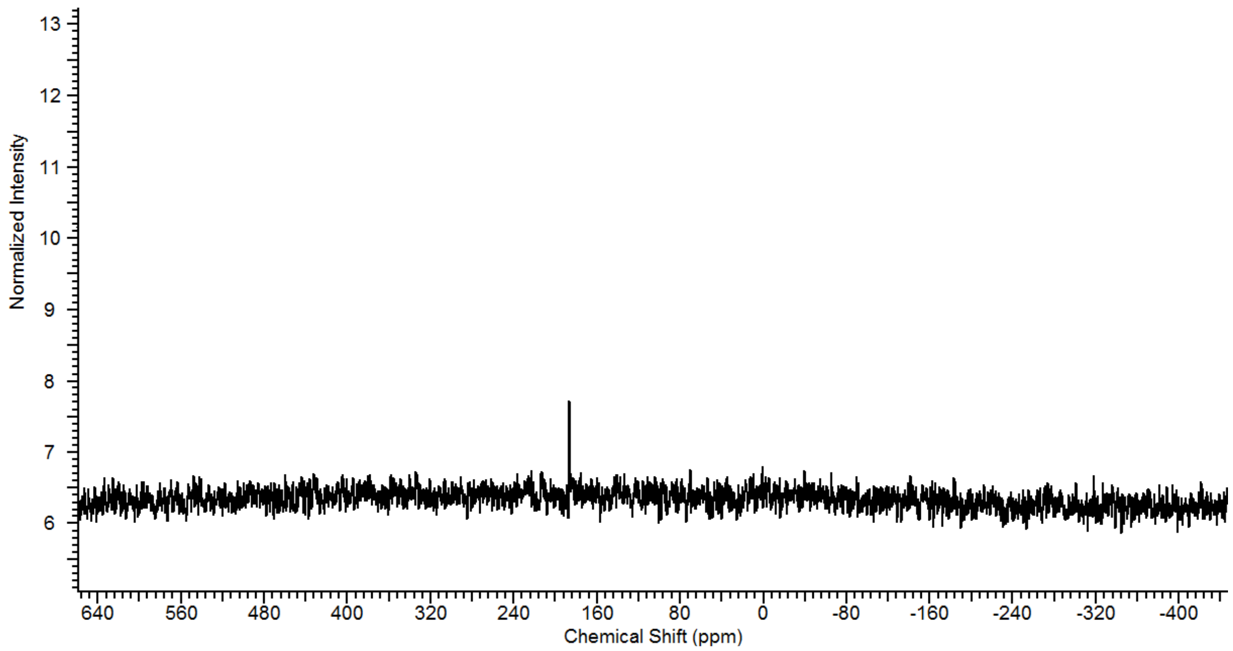

Figure S33. ^{1}H NMR spectrum (401 MHz, $CDCl_3$) of [Fe_2Cp_2(CO)(μ-CO){μ-η^1:η^3-C^3(Ph)C^2(Se)C^1N(Me)(Xyl)}]I, **5b**.

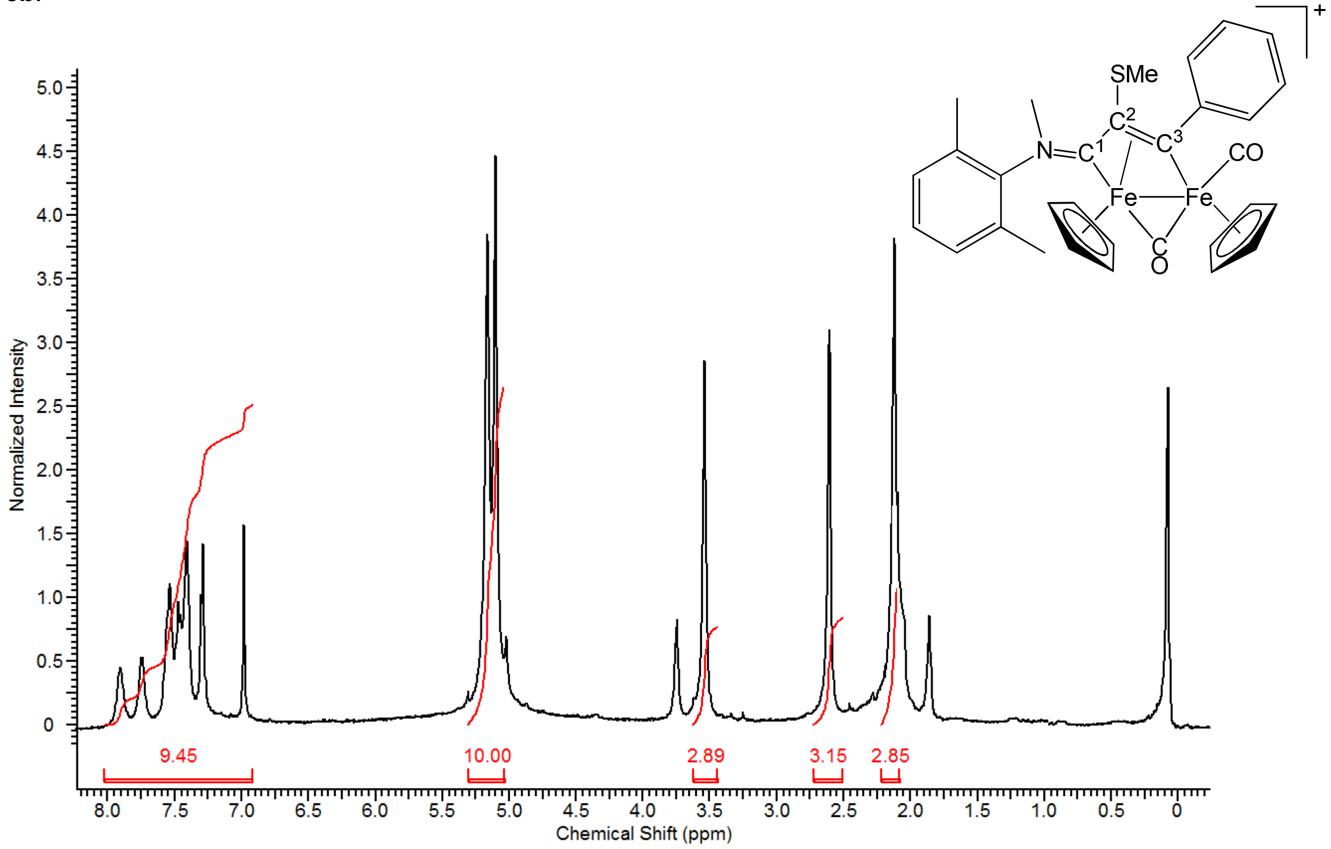

Figure S34. ^{13}C{^{1}H} NMR spectrum (101 MHz, $CDCl_3$) of **5b**.

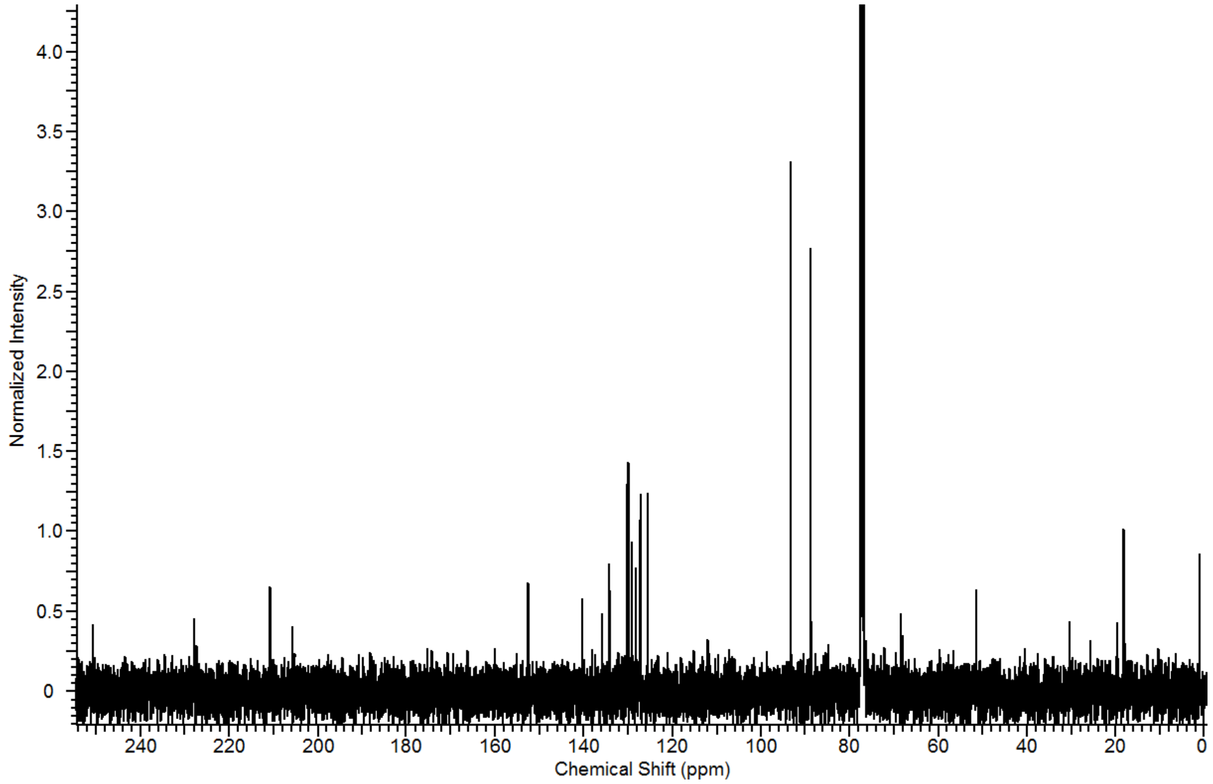

Supplement: Supplementary file 1 [file molecules-25-01656-s001.zip › Supporting Material/Supporting Information.pdf]
